# The Uncommon Phenomenon of Short QT Syndrome: A Scoping Review of the Literature

**DOI:** 10.3390/jpm15030105

**Published:** 2025-03-08

**Authors:** Aristi Boulmpou, Andreas Giannopoulos, Christodoulos Papadopoulos, Georgios Giannopoulos, Ioannis Papagiannis, Georgios Zormpas, Anastasia Keivanidou, Liana Fidani, Vassilios Vassilikos

**Affiliations:** 1 Third Department of Cardiology, Ippokratio General Hospital of Thessaloniki, Aristotle University of Thessaloniki, 54642 Thessaloniki, Greece; 2 Department of Pediatric Cardiology, Second Department of Pediatrics, AHEPA University Hospital, Aristotle University of Thessaloniki, 54636 Thessaloniki, Greece; 3 Department of Pediatric Cardiology and Adult Congenital Heart Disease, Onassis Cardiac Surgery Centre, 17674 Athens, Greece; 4 Second Department of Cardiology, Ippokratio General Hospital of Thessaloniki, Aristotle University of Thessaloniki, 54642 Thessaloniki, Greece; 5 Second Department of Pediatrics, AHEPA University Hospital, Aristotle University of Thessaloniki, 54636 Thessaloniki, Greece

**Keywords:** short QT syndrome, channelopathy, sudden cardiac death, genetic mutations

## Abstract

**Background:** Short QT syndrome (SQTS) is a rare inheritable channelopathy characterized by a shortened corrected QT interval on an electrocardiogram and a significant risk of atrial and ventricular arrhythmias, potentially leading to sudden cardiac death. Despite advancements in our understanding of SQTS, knowledge gaps persist due to its extreme rarity. This scoping review aims to summarize the available knowledge on its clinical presentations, genetic mutations, and management strategies, while identifying areas for further investigation. **Methods:** This scoping review was conducted across the PubMed, Scopus, and Cochrane databases and identified relevant case reports, case series, and available studies on SQTS. We focused on articles that reported clinical outcomes, genetic mutations, diagnostic criteria, and management strategies, while excluding studies on the secondary causes of short QT intervals. **Results:** SQTS is present across a wide age range, from asymptomatic individuals to those experiencing syncope, palpitations, or sudden cardiac arrest. Common genetic mutations include *KCNQ1*, *KCNH2*, and *KCNJ2*. Management strategies vary, with some patients receiving implantable cardioverter defibrillators for secondary prevention and others treated pharmacologically, primarily with hydroquinidine. Our findings highlight the rarity and clinical variability of SQTS, underscoring the need for optimized diagnostic criteria and individualized management strategies. **Conclusions:** This review emphasizes the need for continued research to better understand the genetic basis of SQTS, optimize diagnostic tools, and improve treatment approaches. Large-scale studies and the integration of genetic and clinical data are critical to addressing the gaps in SQTS management and improving outcomes for patients with this potentially life-threatening arrhythmic disorder.

## 1. Introduction

Short QT syndrome (SQTS) is a rare and potentially life-threatening inheritable channelopathy characterized by abbreviated corrected QT (QTc) intervals on electrocardiograms (ECGs). First described in 2000, SQTS has since emerged as a distinct entity associated with increased risk for ventricular arrhythmias, syncope, and sudden cardiac death (SCD) in the absence of structural heart disease [1]. While our understanding of the syndrome has grown significantly over the past 20 years, knowledge gaps persist, especially regarding primary prevention.

**Figure 1:**
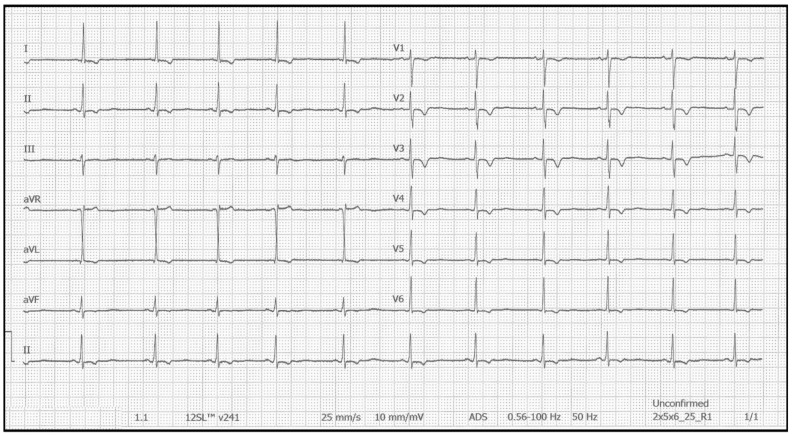
Representative 12-lead ECG from a patient with SQTS, demonstrating a markedly shortened QT interval (QTc: 290 ms) and repolarization abnormalities.

Using the Schwartz criteria for long QT syndrome as an example [2], Gollob et al. proposed diagnostic criteria for SQTS based on ECG and clinical characteristics, family history, and genetic findings [3]. The clinical presentation of the syndrome is highly variable, ranging from asymptomatic individuals to those experiencing palpitations, syncope, or life-threatening arrhythmias, such as ventricular fibrillation (VF). Given this variability, the diagnosis of SQTS can be particularly challenging and often requires a high index of suspicion; the recent European Society of Cardiology (ESC) guidelines for the management of patients with ventricular arrhythmias and the prevention of SCD, published in 2022 [4], included updated diagnostic criteria for SQTS and suggested an implantable cardioverter defibrillator (ICD) for patients who are survivors of cardiac arrest or demonstrate a history of arrhythmic syncope. In terms of pharmaceutical options, hydroquinidine is recommended for the prevention of ventricular arrhythmias in high-risk SQTS patients who do not wish to have an ICD implanted [4]. A representative ECG of a patient with SQTS is shown in Figure 1, demonstrating their short QT (SQT) interval and repolarization abnormalities.

Since the initial description of the syndrome, various gene mutations have been identified in SQTS patients, with seven types reported so far. SQTS 1–3 have been associated with mutations resulting in a gain of function for potassium channels, SQTS 4–6 with mutations causing loss of function for calcium channels, while the seventh subtype of SQTS is linked to a loss of function in the anion exchanger AE3 [5,6]. However, in a large proportion of SQTS cases, genetic testing is negative for known mutations, suggesting the presence of additional genetic contributors that are yet to be identified [4]. The close follow-up of these patients, with periodically repeated genetic testing for newly described mutations, as well as cascade screening and genetic counseling, are of paramount importance for a better understanding of these cases.

In an effort to provide a comprehensive overview of the current state of knowledge on SQTS, we conducted a scoping review of the literature. Our aim was to summarize existing data on the clinical presentation, genetic basis, diagnostic criteria, management options, and outcomes for patients with SQTS; by identifying areas where knowledge is lacking, we hope to inform future research directions, with an eye to improve the treatment approaches for patients suffering from this rare but potentially life-threatening arrhythmic syndrome.

## 2. Materials and Methods

This scoping review of the literature was conducted in accordance with the Preferred Reporting Items for Systematic Reviews and Meta-Analyses guidelines for scoping reviews (PRISMA-ScR) (Supplementary Table S1). We searched the PubMed, Scopus, and Cochrane databases from their inception to January 5th, 2025, employing Medical Subject Heading (MeSH) terms such as “short QT syndrome”, “SQTS”, “short QT”, “QT shortening”, “QTc”, and “SQT”. No restrictions were placed in terms of time, while only English language articles were incorporated. Our search was designed to identify the available case reports, case series, and all other types of studies including patients with SQTS. Systematic reviews, narrative reviews, and expert opinions, as well as journal preprints and animal and cell model studies, were excluded. This scoping review was not registered in PROSPERO or other databases, as its nature is purely exploratory, rather than a systematic review with a predefined protocol.

All retrieved results were imported into reference management software for duplicate removal. Two independent reviewers scanned the results’ titles and abstracts for relevance (AB and AK). Eligible manuscripts then had their full text reviewed, while the PICO (Patients, Interventions, Control and Outcomes) process was used to construct data extraction tables from the retrieved studies. More detailed tables were also created to extract data from case reports and case series. Full-text reports were scanned for further references. A critical appraisal of the retrieved studies was not performed, since the goal of our search was to identify potential parameters of interest, rather than evaluate the level of evidence.

## 3. Results

**Figure 2:**
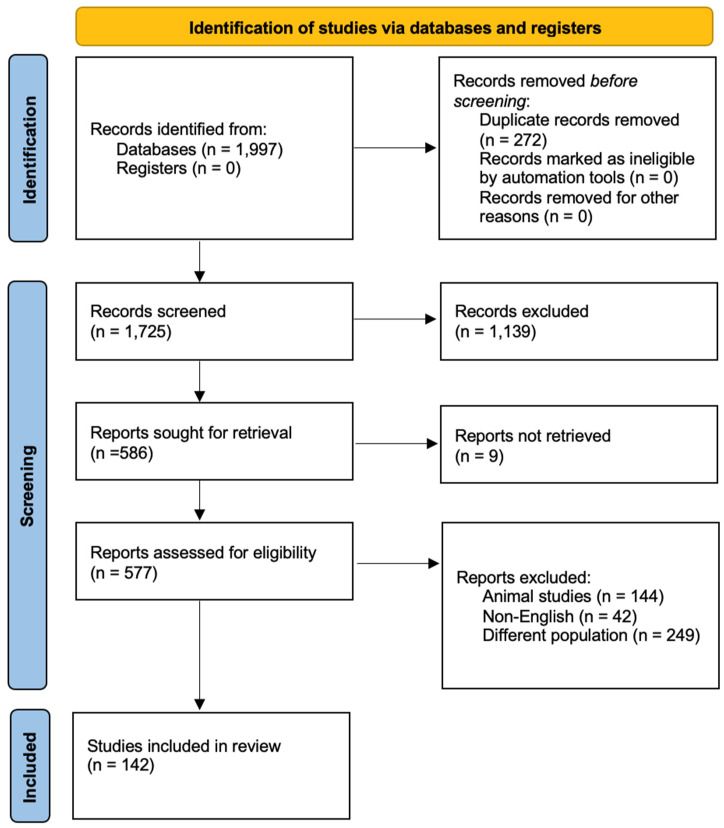
PRISMA 2020 flow diagram illustrating the study selection process. This diagram outlines the identification, screening, eligibility assessment, and final inclusion of studies in this scoping review. A total of 1997 records were identified through database searches, with 273 duplicates then removed. After screening and an eligibility assessment, 141 studies were included in the final review. The exclusion criteria were non-English studies, animal studies, and studies focusing on different populations.

Our search delivered a total of 1997 results, and, after duplicate removal, the titles and abstracts of 1724 references were screened for eligibility. A total of 1139 articles were initially excluded as irrelevant to the topic and, after full-text review, an additional 444 articles were rejected. We therefore included 141 references in our scoping review (Figure 2). In this review, specific emphasis was placed on SQTS, and references pertaining to secondary causes of SQT were deliberately excluded from the analysis in order to maintain a focused investigation on the primary genetic disorder.

### 3.1. Case Reports

Our search yielded 43 case reports on SQTS (Supplementary Table S2) [7,8,9,10,11,12,13,14,15,16,17,18,19,20,21,22,23,24,25,26,27,28,29,30,31,32,33,34,35,36,37,38,39,40,41,42,43,44,45,46,47,48,49], with an average diagnosis age of around 25 years. Ten cases were reported to be asymptomatic; common symptoms included presyncope, syncope, and palpitations, with some cases presenting with aborted cardiac arrest. In total, 15 cases had a positive family history of SCD. Additional ECG abnormalities were observed, such as atrial fibrillation (AF), junctional rhythm, U-waves, ST-segment elevation, and early repolarization (ERP). A variety of diagnostic modalities were implemented for further investigation according to each individual case. Treatment strategies varied, with oral quinidine and b-blockers being commonly used pharmaceutical options and ICD implantation being frequently performed, mainly for secondary prevention. Regarding genetic testing, mutations in the genes *KCNQ1*, *KCNH2*, *SCN5A*, *CACNA1C*, *CACNA2D1*, *KCNJ2*, *SCN10A*, and *SLC22A5* were identified in 19 cases. Genetic testing was not available or not performed in 16 cases and yielded negative results for known mutations or demonstrated variants of unknown significance (VUS) in 8 cases. Two patients had evidence of concomitant hypertrophic cardiomyopathy [18,42] and one patient developed arrhythmogenic ventricular cardiomyopathy [49]. The left ventricular ejection fraction (LVEF) was reported to be reduced in one case [12]. Our search also revealed five case reports describing SQTS in the context of various syndromes or other genetic clinical conditions [10,24,25,28,33].

### 3.2. Case Series

Our review of the literature revealed 29 case series of patients with SQTS [1,5,50,51,52,53,54,55,56,57,58,59,60,61,62,63,64,65,66,67,68,69,70,71,72,73,74,75,76]. In 2000, Gussak et al. [1] presented the first reported family with SQTS, which contained three affected members who demonstrated frequent AF episodes requiring cardioversion. Since then, a series of other affected families have been described, with SQTS manifesting across different age groups. Genetic testing was negative for known mutations in approximately half of these cases; when positive, the identified mutated genes included *KCNQ1*, *KCNJ2*, *KCNH2*, *SLC4A3*, *KCNJ5*, *SCN10A*, *CACNA2D*, and *CACNB2d*. The majority of reported patients presented with a positive family history for SCD, while their symptoms at initial presentation varied from palpitations and dyspnea to SCD. About 16% of the reported patients were asymptomatic.

The largest case series was published by El-Battrawy et al. [58], who described 17 patients within seven unrelated families, followed up with for an average of 13.5 years. SCD occurred in 71% of families, affecting men at a younger age, while AF or atrial flutter occurred in 53% of patients. Interventions included ICD implantation in 29% and oral hydroquinidine in 53% of cases, while the most common mutations identified were located in the *KCNH2* and *CACNB2b* genes. Supplementary Table S3 describes the case series of patients with SQTS retrieved from the literature in more detail.

### 3.3. Studies

A total of 69 studies explored various facets of SQTS, including its prevalence in healthy populations, genetic testing, patient outcomes, drug efficacy, associations with other clinical conditions, and the findings from various diagnostic modalities [52,55,62,65,71,77,78,79,80,81,82,83,84,85,86,87,88,89,90,91,92,93,94,95,96,97,98,99,100,101,102,103,104,105,106,107,108,109,110,111,112,113,114,115,116,117,118,119,120,121,122,123,124,125,126,127,128,129,130,131,132,133,134,135,136,137,138,139,140].

#### 3.3.1. Studies Screening Healthy Populations or Medical Databases for SQTS

Our literature search revealed 22 studies focused on screening healthy individuals or medical databases to detect the presence of SQTS [65,88,89,90,92,93,94,99,103,106,108,114,120,123,125,130,132,133,134,135,139,140]. Iribarren et al. [106] analyzed 6.4 million ECGs, demonstrating a prevalence of very SQT (<300 ms) of 0.7 per 100,000 and a heightened multivariable-adjusted mortality risk (x2.6) over 8 years of follow-up. Estimates of SQT interval prevalence ranging from lower than 1% up to about 7–8% have been reported in different populations [65,93,94,99,108,120,132,140], although the true prevalence of SQTS, in contrast to SQT, is estimated to be less than 1 in 10,000. This is due to the fact that most individuals with a SQT interval do not meet the diagnostic criteria for SQTS. These findings highlight the importance of continued research in order to shed light on the actual prevalence, clinical implications, and optimal management strategies for this rare patient population. Details of the aforementioned studies are presented in Supplementary Table S4.

#### 3.3.2. Genetic Testing in Patients with SQTS

A number of studies have focused on the genetic analysis of SQTS patients [55,62,77,107,115,121,122,136,137]. Brugada et al. [55] identified *KCNH2* mutations in three families with SQTS, linking them to SCD for the first time. *KCNH2* was also implicated by Hu et al. [107], with the identification of a *KCNH2-T618I* mutation associated with SQTS in 18 individuals across seven families, all experiencing SCD. Wu et al. [77] identified the R259H mutation in *KCNQ1* associated with SQTS in the Chinese population. Antzelevitch et al. [137] observed SQT intervals in patients with Brugada syndrome (BrS), alongside their ST-segment elevation and SCD, which were linked to loss-of-function mutations in L-type calcium channel genes. Along the same lines, Burashnikov et al. [136] highlighted L-type calcium channel mutations in patients with BrS and SQTS. Harrell et al. [62] described two novel mutations (*KCNH2*-I560T and *KCNQ1*-V141M) in Japanese families, underlining the diverse clinical manifestations of SQTS. Christiansen et al. [121] indicated potentially disease-causing variants, notably *SLC4A3*, in 26% of patients with SQTS. However, Fukuyama et al. [115] and Blancard et al. [122] did not identify pathogenic variants in their SQTS cohorts.

In short, mutations identified in key genes such as *KCNH2*, *KCNQ1*, and L-type calcium channel genes have been associated with SQTS, although the molecular substrate remains unknown in the majority of cases [4]. In addition, these findings highlight the heterogeneous nature of SQTS and the importance of further investigations into the genetic etiology of the syndrome. Further research is also needed to fully understand its genotype–phenotype interplays and to develop targeted therapies for individuals with SQTS. Supplementary Table S5 presents details on the studies involving the genetic testing of patients with SQTS.

#### 3.3.3. Studies Assessing Outcomes of Patients with SQTS

Studies reporting outcomes in patients with SQTS are summarized in Supplementary Table S6 [80,85,86,95,97,102,110,111,119,129]. Overall, the number of individuals with SQTS included in each study is small, and the reported rates of arrhythmic events attributable to SQTS are relatively low, with a significant proportion of patients experiencing inappropriate shocks and device-related complications [80,84,119,129].

#### 3.3.4. Drug Therapy in Patients with SQTS

Few studies have evaluated the effect of antiarrhythmic medications on patients with SQTS (Supplementary Table S7a) [78,96,113,118,128]. Gaita et al. [113] assessed a series of antiarrhythmic regimens and reported that hydroquinidine prolonged the QT interval, suggesting that this could be a potential alternative or adjunct to ICD implantation. In line with this, quinidine has been shown to suppress IKr function, which may have therapeutic implications for the gain-of-function *HERG* mutations associated with SQTS [78]. Several studies have reported a favorable safety profile for hydroquinidine in SQTS [96,118], with indications of a lower incidence of life-threatening arrhythmias. Schimpf et al. suggested disopyramide as an alternative [128], demonstrating its ability to prolong the QT interval and ventricular effective refractory periods in SQTS patients with specific *HERG* gene mutations.

While available studies indicate that quinidine prolongs the QT interval and may reduce the incidence of ventricular arrhythmias in SQTS patients, this evidence is primarily based on small observational studies with limited follow-up. Disopyramide has also been proposed as an alternative treatment, particularly for those with specific *HERG* gene mutations. However, further research is needed to confirm the long-term efficacy and safety of these pharmacological approaches.

#### 3.3.5. Studies Assessing Families of Sudden Cardiac Death Victims for the Presence of SQTS

Our search of the literature revealed a total of five studies screening families or cases of SCD for the presence of SQTS (Supplementary Table S7b) [71,83,101,105,131]. Wisten et al. [71] analyzed 181 young individuals with normal autopsy findings, identifying one family with SQTS; similarly, only one case of SQTS was found among 35 patients with unexplained cardiac arrest in the cohort study by Jiménez-Jáimez et al. [105]. Stepįeń-Wojno et al. [83] reported SQTS in 7% of 44 screened patients after SCD. Makarov et al. [131] observed a high frequency of SQT intervals in children from families with a history of sudden death; additionally, SQTS was diagnosed in only 1 family among the 109 referred with SCD and 52 probands with unexplained cardiac arrest [101].

Our review suggests a low prevalence of SQTS among individuals with unexplained cardiac arrest or normal autopsy findings, ranging from 1 to 7% in various cohorts. Further studies are needed for a better understanding of the relationship between SQTS and the risk of SCD.

#### 3.3.6. Studies Demonstrating an Association Between SQT Intervals and Other Clinical Conditions

We identified three studies associating SQT intervals with various clinical conditions (Supplementary Table S7c) [100,104,127]. Teh et al. [127] examined 70 epilepsy patients, noting significantly shorter QTc intervals, especially in those with cryptogenic epilepsy, suggesting a possible connection between epilepsy and altered cardiac repolarization. Screening 150 patients with dilated cardiomyopathy for anti-*KCNQ1* antibodies revealed 6% seropositivity and significantly shorter QT intervals [100]. Finally, Jørgensen et al. [104] observed shorter QTc intervals in males with Klinefelter syndrome. Further investigation may elucidate the potential clinical implications of the connection between SQTS and other clinical syndromes.

#### 3.3.7. Findings of Various Diagnostic Modalities Among Patients with SQTS

Eleven studies explored the diagnostic modalities used in patients with SQTS (Supplementary Table S8) [52,79,81,82,85,98,109,116,117,124,138]. Echocardiographic abnormalities, including a reduced LVEF and significant myocardial contraction dispersion, were reported by Frea et al [116]. Exercise testing revealed that these patients had shorter QT intervals both at rest and during peak exercise, with a limited QT adaptation to heart rate [109]. Negative T-wave alternans suggested limited risk stratification [98], while increased transmural dispersion of repolarization was observed through 24-h ECG Holter monitoring [52].

Additional ECG findings included shorter Jpoint-Tpeak intervals in symptomatic patients [138], while Watanabe et al. [79] and Tülümen et al. [81] identified ERP and PQ segment depression, respectively, as potential markers of SQTS-associated arrhythmic events. Suzuki et al. [82] highlighted the utility of Jpoint-Tpeak intervals in children, while Extramiana et al. [117] recommended patient-specific QT correction formulas over Bazett’s. In summary, echocardiography, exercise testing, 24-h ECG Holter monitoring, and ECG markers (e.g., ERP, PQ depression, Jpoint-Tpeak intervals) offer valuable diagnostic insights in SQTS patients.

#### 3.3.8. Other Advances in Research on SQTS

Our search revealed four additional studies involving patients with SQTS [87,91,112,126]. Viskin et al. [126] reported significantly shorter QTc in males with idiopathic VF compared to healthy males (371 ± 22 ms vs. 385 ± 19 ms, *p* = 0.034), with no such differences observed in females. Rollin et al. [87] highlighted shorter atrial and ventricular repolarization refractory periods during EP studies, aiding in the differentiation of SQTS from normal cases. Garvey et al. [112] evaluated cardiac sympathetic denervation in eight children with life-threatening ventricular arrhythmias (one with SQTS), noting significant improvement and reduced ICD discharges in treated patients. Finally, Pasero et al. [91] explored artificial intelligence (AI) algorithms in ECG analysis and found shallow neural networks effective in identifying SQTS patients less likely to experience life-threatening arrhythmias, paving the way for AI-based risk stratification. The aforementioned studies are presented in Supplementary Table S9.

### 3.4. Summary of Findings

Given the heterogeneity of the reported studies, we summarized the key clinical and management-related aspects of SQTS, including patient demographics, diagnostic criteria, treatment strategies, and outcomes. Table 1 provides an overview of the main findings derived from the reviewed reports, highlighting the complexity of diagnosis, risk stratification, and management approaches in SQTS.

## 4. Discussion

The findings of our scoping review underline the high complexity and rarity of SQTS, a condition that poses significant diagnostic and management challenges to clinicians due to its diverse clinical presentations, underlying genetic heterogeneity, and associated risk of SCD. Notably, these aspects have also been described in younger populations with SQTS, further emphasizing the importance of early recognition and risk stratification [141]. SQTS presents with diverse phenotypes, from asymptomatic to SCD, spanning infancy to late adulthood; the threshold for diagnosis can vary, making it essential to have a high index of suspicion, particularly in individuals with a family history of SCD or unexplained syncope. At the same time, beyond SQT interval changes, various accompanying ECG alterations have been described, such as ERP, tall/peaked T waves, ST-segment deviations, and other concomitant channelopathies [142]. AF is common in patients with SQTS, with prevalence estimates ranging between 30% and 60% across various studies, depending on the size and genetic background of the cohort [63,68,88]. SQTS often coexists with cardiomyopathies or systemic syndromes, adding complexity to its diagnosis and management [143]. This variability in clinical presentation, from asymptomatic individuals to those experiencing life-threatening arrhythmias such as VF, further complicates the diagnostic process and necessitates a thorough and multifaceted approach.

An extremely low prevalence of SQTS and high mortality rate have previously been reported, although the true incidence of SCD throughout the spectrum of SQTS remains difficult to assess. As such, while ESC guidelines recommend ICD implantation for patients who are survivors of cardiac arrest or have experienced arrhythmic syncope, the role of ICDs in asymptomatic patients with SQTS remains controversial, given the risks associated with device implantation and the significantly high possibility of inappropriate shocks. Concerning pharmacological therapy, quinidine is promising in prolonging QT intervals and reducing arrhythmias, but its use requires caution due to its potentially serious side effects (e.g., gastrointestinal disorders, cinchonism, and hepatotoxicity) [144]. Other antiarrhythmic drugs have been tested with variable success, and the optimal pharmacological management of SQTS remains an area of active investigation, significantly hampered by the small numbers of affected individuals. Further research is also warranted to establish the clinical significance of the associations between SQT intervals and other clinical syndromes such as cardiomyopathies, which could potentially influence the diagnosis and management of SQTS.

Genetic testing serves as a valuable tool for shedding light on the molecular mechanisms underlying SQTS, identifying mutations in genes like *KCNH2*, *KCNJ2*, and *KCNQ1*, which result in a gain of function for potassium channels, leading to accelerated repolarization of the cardiac action potential. Less commonly, mutations in *CACNA1C*, *CACNB2*, *CACNA2D1* (calcium channel genes), and *SLC4A3* (anion exchanger gene) have been identified. However, less than half of all SQTS cases can be attributed to known mutations, highlighting the need for future research to elucidate the genetic basis of the syndrome. Based on current evidence, only a subset of genes have been confirmed to be strongly associated with SQTS; according to the Clinical Genome Resource (ClinGen), genes such as *KCNH2*, *KCNQ1*, *KCNJ2*, and *SLC4A3* are considered to have sufficient evidence for their inclusion in genetic testing panels. In contrast, genes like *CACNA1c*, *CACNB2b*, and *CACNA2D1* have limited or disputed evidence regarding their role in SQTS, indicating the need for further validation before their routine inclusion in diagnostic evaluations (ClinGen, www.clinicalgenome.org, accessed on 26 January 2025). While our review focuses on gene–disease associations in SQTS, we acknowledge that variant classification follows the ACMG 5-tier system [145], with several mutations in *KCNH2*, *KCNQ1*, *KCNJ2*, and *SLC4A3* being classified as pathogenic or likely pathogenic in databases such as ClinVar and ClinGen. Diagnostic modalities such as echocardiography, exercise testing, and ECG monitoring may aid in its comprehensive assessment, though limitations such as small sample sizes and diagnostic variability must be acknowledged.

We purposefully excluded references pertaining to secondary causes of SQT from the present scoping review. However, secondary causes of SQT, including electrolyte imbalances, drug-induced effects, or other genetic conditions (e.g., primary carnitine deficiency) [146,147,148], should be taken into consideration by clinicians encountering patients with SQT.

Despite the comprehensive nature of our review, certain limitations must be acknowledged. First, the rarity of SQTS and small sample sizes in the majority of the reported studies pose significant challenges, limiting our ability to draw safe conclusions. Updated ESC criteria for the diagnosis of SQTS [4] offer some guidance, but advancements in medical care over the past few decades may also impact reported outcomes, making it difficult to attribute these changes solely to the natural course of the disease. Second, this review does not systematically reassess the pathogenicity classification of all reported variants based on ACMG criteria, as this would require a structured genetic database analysis beyond the scope of a scoping review. Evolving medical technologies and genetic testing methods, as well as AI, could definitely impact the reported outcomes.

In this context, several key areas for future research can be suggested. Large, multicenter registries of SQTS patients are needed to collect comprehensive data on its clinical presentation, genetic mutations, management strategies, and outcomes. Such registries would provide valuable insights into the natural history of SQTS and help identify predictors of adverse events. As a next step, one could highlight the need for randomized controlled trials to evaluate the efficacy and safety of different pharmacological therapies in SQTS patients. Finally, advances in genetic research could allow us to explore new mutations associated with SQTS and provide a better understanding of the molecular mechanisms underlying the syndrome. Integrating genetic data with clinical phenotypes could lead to more personalized approaches to SQTS management.

In conclusion, SQTS is a rare but serious condition with significant implications for affected individuals. This scoping review highlights our current understanding of SQTS, emphasizing the importance of accurate diagnosis, risk stratification, and individualized management. Despite significant advances, many questions remain unanswered, and the need for continued research to improve the outcomes for patients with this uncommon but potentially life-threatening syndrome is of paramount importance.

## Figures and Tables

**Table 1:** Summary of key findings from studies on short QT syndrome.

Parameter	Summary of Findings
Age at diagnosis	Mean age: ~25 years (range: infancy to late adulthood) [1,7,8,9,10,11,12,13,14,15,16,17,18,19,20,21,22,23,24,25,26,27,28,29,30,31,32,33,34,35,36,37,38,39,40,41,42,43,44,45,46,47,48,49,93,108,120,141,142]
Gender distribution	SQTS has been reported in both males and females, though some studies suggest a slight male predominance (Supplementary Tables S2 and S3, [104,126,133])
Diagnostic criteria	QTc ≤ 360 ms and pathogenic mutation or family history of SQTS or survival of VT/VF in the absence of heart disease; diagnosis should be considered when QTc is less than 320 ms, or in the presence of QTc >= 320 ms and =< 360 ms and arrhythmic syncope or a family history of SD at an age younger than 40 years [4]
ICD implantation	Recommended for survivors of cardiac arrest or for patients with documented sustained VT or those with arrhythmic syncope; variable use in asymptomatic cases [3,4,80,112,119,126,129,143]
Pharmacological therapy	Hydroquinidine (most studied drug) is effective in QT prolongation and arrhythmia suppression; other drugs (e.g., sotalol, flecainide) have had variable success [3,4,78,113,143]
Outcomes	High risk of arrhythmic events, with some studies reporting inappropriate ICD shocks (up to 64% of ICD recipients); limited long-term survival data [1,3,4,65,106,108,112,120,141,142]
Shock-free survival	Median follow-up suggests high incidence of ICD interventions, including both appropriate and inappropriate shocks [4,65,80,93,106,108,119,120,131,143]
Genetic testing	Most common mutations: *KCNH2*, *KCNJ2*, and *KCNQ1* (ClinGen-approved); weaker evidence of mutation in other genes (e.g., *CACNA1C*, *CACNB2*, *CACNA2D1*) [3,4,55,62,77,100,115,121,122,136,137]
Associated conditions	SQTS often coexists with cardiomyopathies, early repolarization syndrome, and other inherited arrhythmias [18,42,49,54,73,95,100,142]
Future directions	Need for multicenter registries, genetic studies, and RCTs to refine risk stratification and treatment approaches

Abbreviations: ICD, implantable cardioverter defibrillator; QTc, corrected QT; SD, sudden death; SQTS, short QT syndrome; VF, ventricular fibrillation; VT, ventricular tachycardia.

## Data Availability

No new data were created or analyzed in this study.

## Supplementary Materials

The following supporting information can be downloaded at: https://www.mdpi.com/article/10.3390/jpm15030105/s1, Table S1: Preferred Reporting Items for Systematic reviews and Meta-Analyses extension for Scoping Reviews (PRISMA-ScR) Checklist; Table S2: Case reports comprising patients with short QT syndrome; Table S3: Table presenting available case series involving families or distinct cases with short QT syndrome; Table S4: Studies assessing healthy populations or health databases for the presence of short QT syndrome; Table S5: Studies implementing genetic testing in patients with short QT syndrome; Table S6: Studies assessing outcomes in patients with short QT syndrome; Table S7a: Studies assessing the effects of pharmaceutical interventions among patients with short QT syndrome; Table S7b: Studies comprising identification of short QT syndrome cases among families with history of sudden cardiac arrest or sudden cardiac death; Table S7c: Studies demonstrating association of short QT with other clinical conditions; Table S8: Studies involving implementation of various diagnostic modalities among patients with short QT syndrome; Table S9: Studies involving other advances in research regarding short QT syndrome.

## Abbreviations

The following abbreviations are used in this manuscript:

SQTS	Short QT syndrome
QTc	Corrected QT
ECG	Electrocardiogram
SCD	Sudden cardiac death
VF	Ventricular fibrillation
ESC	European Society of Cardiology
ICD	Implantable cardioverter defibrillator
SQT	Short QT
AF	Atrial fibrillation
ERP	Early repolarization
LVEF	Left ventricular ejection fraction
BrS	Brugada syndrome
AI	Artificial intelligence
