# Supplementary material for: The Uncommon Phenomenon of Short QT Syndrome: A Scoping Review of the Literature
Source: J Pers Med. 2025 Mar 8;15(3):105. doi: 10.3390/jpm15030105 (PMC11943495; doi:10.3390/jpm15030105)
Supplement: Supplementary file 1 [file jpm-15-00105-s001.zip › Supplementary Table S2 - Case reports OK.pdf]

Supplementary Table S2. Case reports comprising patients with short QT syndrome.

Reference	Age	Gender	Presentation	Family history of SCD	ECG (QTc)	Concomitant ECG changes	Genetic testing	Other diagnostic investigations	Treatment/management	Comments
**Bellocq et al, 2004 [41]**	70y	M	Aborted CA (VF)	N	302	-	g919c substitution in KCNQ1	TTE, exercise testing, laboratory testing, coronary angiography, scintigraphy, ergonovine coronary spasm test, EPS, LV myocardial biopsies	N/A	-
**Kirilmaz et al, 2005 [39]**	20y	M	Exertional dyspnea	N	257 - 302 (Bazzett)	• AF<br>• Sinus bradycardia<br>• U-waves in precordial leads	Pending until the date of publication	Chest x-ray, TTE, laboratory testing, exercise test, EPS	Anticoagulation, quinidine	-
**Schimpf et al, 2005 [38]**	15y	M	N/A	Y	252	N/A	N/A	EPS	ICD implantation	Appropriate ICD shock due to VF
**Hong et al, 2005 [40]**	Newborn	F	Fetal bradycardia, AF	N/A	N/A	N/A	V141M mutation in KCNQ1	Chest x-ray, laboratory testing, EPS, TEE	Unsuccessful electrical cardioversion of AF	-
**Morphet et al, 2007 [47]**	3w	N/A	Apnea	N/A	210	• Wandering pacemaker<br>• Sinoatrial exit block	N/A	N/A	Critical SND and death	-
**Fichet et al, 2008 [37]**	21y	F	Aborted CA (VF)	N	315 (Bazzett)	Peaked T waves	N/A	Laboratory testing, blood toxicologic screening	N/A	Death after unsuccessful electrical cardioversion of VF
**Mizobuchi et al, 2008 [36]**	24y	M	Syncope	Y	308 (Bazzett)	N/A	Negative for known mutations	TTE, exercise testing, CMR, cardiac CT, laboratory testing, EPS	ICD implantation	Drug challenge during EPS using nifekalant and disopyramide
**Efremidis et al, 2009 [35]**	17y	N/A	Syncope	N	283 (Bazzett), 295 (Fridericia), 288 (Framingham)	• Prominent J-waves<br>• ST segment elevation<br>• Paroxysmal A-V block	KCNQ1 (1638 G > A S546S), KCNH2 (G3152+22G> A) and SCN5A	TTE, exercise test, tilt test, 24h ECG Holter monitoring, procainamide testing, EPS	Quinidine, ICD implantation	-

							(5457C>TD1819D, 3183 G >A E1061E)			
**Itoh et al, 2009 [34]**	34y	M	Asymptomatic	N/A	329	Brugada-type 2 ECG	R1135H mutation in KCNH2	24h ECG Holter monitoring, TTE, CMR, EPS	ICD implantation	-
**Rooryck et al, 2009 [33]**	3y 8mo	F	Psychomotor development, cognitive impairment, facial dysmorphic features	N	330	N/A	12p13.33 deletion in CACNA1C	TTE	N/A	SQTS in the context of systemic syndrome
**Villafane et al, 2009 [32]**	13y	M	Aborted CA during exercise	N	319	• Symmetric, tall and narrow T-waves<br>• Absence of ST segment	Pending until the date of publications	Chest x-ray, laboratory testing, 24h ECG Holter monitoring, EPS	Quinidine, ICD implantation	-
**Sun et al, 2010 [31]**	45y	M	Occasional dizziness	Y	298	High-peaked T-waves	N/A	Chest x-ray, laboratory testing, EPS	ICD implantation	Inappropriate shocks due to T-wave oversensing
**Templin et al, 2011 [30]**	17y	F	Aborted CA	N/A	329	N/A	c.2264G>C mutation in CACNA2D1	TTE, coronary angiography, EPS	Oral nebivolol, ICD implantation	-
**Zienciuk-Krajka et al, 2012 [42]**	22y	M	First episode of AF	N	280 (QT)	N/A	Negative for known mutations	Laboratory testing, 24h ECG Holter monitoring, TTE, EPS	Pharmacological cardioversion to SR	Asymmetric IVS hypertrophy
**Hattori et al, 2012 [28]**	8y	F	Mental retardation, abnormal proliferation of esophageal blood vessels, epilepsy, Kawasaki disease	Y	194	AF	M301K mutation in KCNJ2	TTE, RHC	Electrical cardioversion	SQTS in the context of systemic syndrome

**Chinushi et al, 2012 [29]**	15y	M	Asymptomatic	Y	338 (Bazzett), 286 (Fridericia)	Peaked T-waves	C5457T polymorphism at SCN5A	Exercise test, laboratory testing, 24h ECG Holter monitoring, cardiac catheterization, EPS	None (patient and parents denied ICD implantation)	-
**Hong et al, 2012 [27]**	40y	M	Asymptomatic	Y	320 (QT)	Brugada-like ECG	R689H mutation in SCN5A	TTE, laboratory testing	Refused EPS	-
**Deo et al, 2013 [26]**	11y	M	PAF, mild LV dysfunction	N	200 (QT)	• QRS merged with T-wave<br>• Absent ST segment in all leads<br>• Peaked T-waves	E299V mutation in KCNJ2	Exercise test, TTE, 24h ECG Holter monitoring, CMR	Cardioversion to SR, oral amiodarone, oral b-blockers	-
**Portugal et al, 2014 [46]**	52y	M	Syncope	Y	329 (Bazzett)	N/A	Negative for known mutations	Laboratory testing, cardiac catheterization, TTE, 24h ECG Holter monitoring, EPS	Oral quinidine, ICD implantation	Polymorphic VT during monitoring requiring electrical cardioversion
**Jeppensen et al, 2014 [25]**	25y	M	Therapy-resistant focal epilepsy, presurgical evaluation	N	335 – 358	N/A	N/A	HRV analysis	CA and death after generalized tonic-clonic seizures	-
**Maltret et al, 2014 [24]**	Newborn	F	Fetal bradycardia	N/A	279 (Bazzett), 272 (Fridericia)	N/A	V141M mutation in KCNQ1	24h ECG Holter monitoring	Hydroquinidine	Concomitant nystagmus, no hearing deficiency
**Pavão et al, 2014 [23]**	23y	M	Palpitations, aborted CA during intense physical activity (soccer)	N	349 (Framingham)	ERP in inferior leads	N/A	Exercise test, 24h ECG Holter monitoring, TTE, CMR, cardiac catheterization, EPS	ICD implantation	CA during intense exercise
**Sadeghian et al, 2014 [22]**	28y	M	Palpitations, wide QRS complex tachycardia	N/A	<300	Absent ST-segment	N/A	Laboratory testing, EPS	VT ablation	-
**Ergül et al, 2015 [21]**	14y	M	Syncope	Y	320	ERP	Pending until the	Laboratory testing, chest x-ray, TTE, EPS	ICD implantation, oral sotalol	-

							date of publication			
**Righi et al, 2016 [20]**	Newborn	F	Asymptomatic	N/A	270 - 310	• Sinus bradycardia<br>• Sinus pauses (3,1 sec)	V141M mutation in KCNQ1	EPS, TEE	Pacemaker implantation, oral anticoagulation, hydroquinidine, sotalol, amiodarone	AF at the age of 16y
**Sharma et al, 2016 [19]**	10y	M	Aborted CA	N	305	Short J-point to T-peak interval	N/A	Laboratory testing, brain MRI, electroencephalography	ICD implantation	-
**Chen et al, 2017 [18]**	52y	M	Chest discomfort, stress-induced dyspnea	Y	356	• LBBB<br>• ERP in inferior-lateral leads<br>• ST elevation in V4-V6<br>• AF	Trigenic mutations of p.E234K in the DES, p.R989H in the MYPN, and p.R1973P in CACNA1C	TTE	Metoprolol, refused alcohol septal ablation, CRT-D implantation performed	Concomitant obstructive HCM
**Terlemez et al, 2018 [44]**	5y	F	Asymptomatic	Y	340	Narrow and sharp T-waves	Negative for known mutations	24h ECG Holter monitoring	Oral sotalol	-
**Di Stolfo et al, 2018 [16]**	47y	F	Aborted CA	Y	303	• ST elevation in leads I – II – III, V3-V6<br>• Brugada-like pattern<br>• Long PR (280 ms)<br>• ERP	SCN10A mutation	Coronary angiography, laboratory testing, TTE, cranial CT scan, toxicological tests	N/A	Death for reported neurological injury
**Binda et al, 2018 [17]**	23y	M	Asymptomatic	N	320	N/A	c.173T>C mutation in KCNJ2	N/A	N/A	Concomitant ASD
**Wakatsuki et al, 2018 [15]**	22y	M	Aborted CA	N	340	PQ depression	W927G mutation in KCNH2	TTE, coronary angiography, laboratory testing, 24h ECG Holter monitoring	ICD implantation	CA during intense exercise
**Spartalis et al, 2019 [45]**	14y	M	Syncope	N/A	320	Negative for known mutations	N/A	CMR, coronary angiography, EPS	ICD implantation, oral quinidine	Electrical storm treated with oral quinidine

**Farag et al, 2019 [48]**	7m	M	Persistent 2:1 tachycardia	N	246	N/A	N/A	Diagnosis of SQTS during EPS	External automatic defibrillator initially, ICD later after aborted CA	Reproducible susceptibility for VF treated with topical lidocaine during ICD implantation
**Guo et al, 2019 [14]**	54y	M	N/A	Y	298	High peaked T-waves in precordial leads	T618I mutation in KCNH2	N/A	ICD implantation	-
**Morimoto et al, 2019 [13]**	50y	F	Aborted CA	Y	308 (Bazzett)	N/A	Negative for known mutations	TTE, coronary angiography, CMR, laboratory testing, EPS	ICD implantation	Polymorphic VT and VF after ICD implantation → PVC ablation, failure of quinidine and sotalol to normalize QT
**Pugliese et al, 2019 [12]**	72y	F	Syncope	N/A	310	• QS in leads V1-V3<br>• ST elevation in leads V1-V4<br>• Repolarization abnormalities and evidence of LVH in leads I-aVL<br>• Small U-waves	Not performed	TTE, laboratory testing, coronary angiography, ventriculography, 24h ECG Holter monitoring	ICD implantation	HFrEF of ischemic etiology (previous myocardial infarction)
**Shen et al, 2019 [11]**	54y	M	N/A	Y	N/A	N/A	N/A	N/A	ICD implantation	Inappropriate therapies due to T-wave oversensing
**Endres et al, 2020 [10]**	34y	M	Whole spectrum of ASD symptoms	N	324 ms	N/A	c.2399A>C mutation in CACNA1C	Electroencephalography, 24h ECG Holter monitoring, TTE,	Quinidine, RFA of PVCs	Symptoms overlapping with Timothy syndrome
**Ramoglu et al, 2020 [9]**	14y	M	Aborted CA	N/A	320	N/A	Negative for known mutations	Laboratory testing, TTE, 24h ECG Holter monitoring	ICD implantation, quinidine	CA during intense exercise

**Chevalier et al, 2021 [49]**	34y	M	Syncope	Y	338 (Bazzett)	None	Negative for known mutations	TTE, cardiac CT, SPECT angioscintigraphy, exercise testing	ICD implantation, oral nadolol, HF treatment, heart transplantation	VF treated with external cardioversion, progression to ARVC
**Grytsay et al, 2022 [8]**	N/A	N/A	Syncope	N/A	<320	N/A	N/A	TTE	N/A	-
**Ploneda-Valencia et al, 2022 [7]**	16y	M	Palpitations, dyspnea, dizziness	N	340	Junctional rhythm	5 VUS	N/A	Propafenone	Atrial flutter episode
**Van Schie et al, 2023 [43]**	14m	F	Chronotropic incompetence	N	285	Changes in T-wave morphology	c.421G>A mutation in KCNQ1 gene	24h ECG Holter monitoring, TTE, EPS	Oral aspirin, PCM implantation (VVI mode) due to nodal rhythm and persistent AF	Concomitant PDA, gradual LV systolic and diastolic function deterioration

Abbreviations: ABG, arterial blood gas; AF, atrial fibrillation; ASD, autism spectrum disorder; ARVC, arrhythmogenic right ventricular cardiomyopathy; CA, cardiac arrest; CMR, cardiovascular magnetic resonance; CRT-D, cardiac resynchronization therapy-defibrillator; CT, computed tomography; ECG, electrocardiogram; EPS, electrophysiological study; ERP, early repolarization; HF, heart failure; HFmrEF, heart failure with mildly reduced ejection fraction; HFrEF, heart failure with reduced ejection fraction; HCM, hypertrophic cardiomyopathy; HRV, heart rate variability; ICD, implantable cardioverter defibrillator; ILR, implantable loop recorder; IVS, interventricular septum; LBBB, left bundle branch block; LMWH, low molecular weight heparin; LV, left ventricular; MRI, magnetic resonance imaging; PCM, pacemaker; PDA, patent ductus arteriosus; PVC, premature ventricular contraction; RFA, radiofrequency ablation; RHC, right heart catheterization; SCD, sudden cardiac death; SND, sinus node dysfunction; SOB, shortness of breath; SPECT, single photon emission computed tomography; SQTS, short QT syndrome; SR, sinus rhythm; TEE, transesophageal echocardiogram; TTE, transthoracic echocardiogram; VF, ventricular fibrillation; VT, ventricular tachycardia; VUS, variant of unknown significance.

References

7. Ploneda-Valencia, R.G.; Ortiz-Solis, W.A.; Ruiz-Gonzalez, G.; Santiago-Garcia, A.K.; Rivera-Rodríguez, L.; Nava-Townsend, S.; Márquez, M.F.; Levinstein-Jacinto, M. Supraventricular tachyarrhythmia and sinus node dysfunction as a first manifestation of short QT syndrome in a pediatric patient. Case Report. *J. Electrocardiol*. **2022**, *74*, 146–153.
8. Grytsay, O.N.; Skybchyk, Y.V.; Shorikova, D.V.; Shorikov, E.I. Clinical Cases of Life—Threatening Arrhythmias: Long and Short Qt Syndromes. *Wiad. Lek.* **2022**, *75*, 1805–1812.
9. Ramoğlu, M.G.; Karagözlü, S.; Uçar, T.; Tutar, E. Aborted cardiac arrest during sport activity in a teenager diagnosed with short QT syndrome. *Cardiol. Young*. **2020**, *30*, 886–889.
10. Endres, D.; Decher, N.; Röhr, I.; Vowinkel, K.; Domschke, K.; Komlosi, K.; Tzschach, A.; Gläser, B.; Schiele, M.A.; Runge, K.; et al. New Cav1.2 Channelopathy with High-Functioning Autism, Affective Disorder, Severe Dental Enamel Defects, a Short QT Interval, and a Novel CACNA1C Loss-Of-Function Mutation. *Int. J. Mol. Sci.* **2020**, *21*, 8611.
11. Shen, Y.; Pan, W.; Jiang, C.; Fu, G.; Sun, Y.; Hu, D. Implantable cardioverter defibrillator replacement guided by T wave safety margin in a short QT syndrome patient. *Pacing Clin. Electrophysiol.* **2019**, *42*, 557–559.
12. Pugliese, D.N.; Reiffel, J.A. Implantable Cardioverter-defibrillator Therapy for Syncope: An Educational Example of a Multicomponent Electrocardiographic Differential Diagnosis and the Application of Clinical Trial Data to an Individual Patient. *J. Innov. Card. Rhythm. Manag.* **2019**, *10*, 3860–3864.
13. Morimoto, Y.; Watanabe, A.; Morita, H.; Nishii, N.; Nakamura, K.; Ito, H. Successful radiofrequency catheter ablation of a premature ventricular contraction triggering ventricular fibrillation in a patient with short QT syndrome. *Hear. Case Rep.* **2019**, *5*, 262–265.
14. Guo, F.; Sun, Y.; Wang, X.; Wang, H.; Wang, J.; Gong, T.; Chen, X.; Zhang, P.; Su, L.; Fu, G.; et al. Patient-Specific and Gene-Corrected Induced Pluripotent Stem Cell-Derived Cardiomyocytes Elucidate Single-Cell Phenotype of Short QT Syndrome. *Circ. Res.* **2019**, *124*, 66–78.
15. Wakatsuki, D.; Iso, Y.; Mase, H.; Kurata, M.; Kyuno, E.; Shimojima, H.; Asano, T.; Sambe, T.; Suzuki, H. Sudden cardiac arrest during marathon training in a young adult with short QT syndrome. *Int. J. Cardiol. Heart Vasc.* **2018**, *18*, 101–103.
16. Di Stolfo, G.; Palumbo, P.; Castellana, S.; Mastroianno, S.; Biagini, T.; Palumbo, O.; Leone, M.P.; De Luca, G.; Potenza, D.R.; Mazza, T.; et al. Sudden cardiac death in J wave syndrome with short QT associated to a novel mutation in Na(v) 1.8 coding gene SCN10A: First case report for a possible pharmacogenomic role. *J. Electrocardiol.* **2018**, *51*, 809–813.
17. Binda, A.; Rivolta, I.; Villa, C.; Chisci, E.; Beghi, M.; Cornaggia, C.M.; Giovannoni, R.; Combi, R. A Novel KCNJ2 Mutation Identified in an Autistic Proband Affects the Single Channel Properties of Kir2.1. *Front. Cell Neurosci.* **2018**, *12*, 76.
18. Chen, Y.; Barajas-Martinez, H.; Zhu, D.; Wang, X.; Chen, C.; Zhuang, R.; Shi, J.; Wu, X.; Tao, Y.; Jin, W.; et al. Novel trigenic CACNA1C/DES/MYPN mutations in a family of hypertrophic cardiomyopathy with early repolarization and short QT syndrome. *J. Transl. Med.* **2017**, *15*, 78.
19. Sharma, P.K.; Awasthy, N. Bystander Cardio Pulmonary Resuscitation Saves Life in a Patient with Short QT Syndrome. *Indian Pediatr.* **2016**, *53*, 933–934.
20. Righi, D.; Silvetti, M.S.; Drago, F. Sinus bradycardia, junctional rhythm, and low-rate atrial fibrillation in Short QT syndrome during 20 years of follow-up: Three faces of the same genetic problem. *Cardiol. Young.* **2016**, *26*, 589–592.
21. Ergül, Y.; Özyılmaz, İ.; Onan, S.H.; Güzeltaş, A. Short QT syndrome in a 14-year-old patient: The first pediatric case from Turkey. *Anatol. J. Cardiol.* **2015**, *15*, 590–591.
22. Sadeghian, S.; Bozorgi, A.; Safkhani, Z. Short QT syndrome and idiopathic ventricular tachycardia in a 28-year-old young man: A potential disease-specific link? *Europace* **2014**, *16*, 1645.
23. Pavão, M.L.; Ono, V.C.; Arfelli, E.; Simões, M.V.; Marin Neto, J.A.; Schmidt, A. Sudden cardiac death and short QT syndrome. *Arq. Bras. Cardiol.* **2014**, *103*, e37–e40.
24. Maltret, A.; Wiener-Vacher, S.; Denis, C.; Extramiana, F.; Morisseau-Durand, M.P.; Fressart, V.; Bonnet, D.; Chabbert, C. Type 2 short QT syndrome and vestibular dysfunction: Mirror of the Jervell and Lange-Nielsen syndrome*? Int. J. Cardiol.* **2014**, *171*, 291–293.
25. Jeppesen, J.; Fuglsang-Frederiksen, A.; Brugada, R.; Pedersen, B.; Rubboli, G.; Johansen, P.; Beniczky, S. Heart rate variability analysis indicates preictal parasympathetic overdrive preceding seizure-induced cardiac dysrhythmias leading to sudden unexpected death in a patient with epilepsy. *Epilepsia* **2014**, *55*, e67–e71.
26. Deo, M.; Ruan, Y.; Pandit, S.V.; Shah, K.; Berenfeld, O.; Blaufox, A.; Cerrone, M.; Noujaim, S.F.; Denegri, M.; Jalife, J.; et al. KCNJ2 mutation in short QT syndrome 3 results in atrial fibrillation and ventricular proarrhythmia. *Proc Natl Acad Sci USA* **2013**, *110*, 4291–4296.
27. Hong, K.; Hu, J.; Yu, J.; Brugada, R. Concomitant Brugada-like and short QT electrocardiogram linked to SCN5A mutation. *Eur. J. Hum. Genet.* **2012**, *20*, 1189–1192.
28. Hattori, T.; Makiyama, T.; Akao, M.; Ehara, E.; Ohno, S.; Iguchi, M.; Nishio, Y.; Sasaki, K.; Itoh, H.; Yokode, M.; et al. A novel gain-of-function KCNJ2 mutation associated with short-QT syndrome impairs inward rectification of Kir2.1 currents. *Cardiovasc. Res.* **2012**, *93*, 666–673.

29. Chinushi, M.; Sato, A.; Iijima, K.; Suzuki, K.; Hiroshi, F.; Izumi, D.; Watanabe, H.; Kanae, H.; Aizawa, Y. Exercise-related QT interval shortening with a peaked T wave in a healthy boy with a family history of sudden cardiac death. *Pacing Clin. Electrophysiol.* **2012**, *35*, e239–e242.
30. Templin, C.; Ghadri, J.R.; Rougier, J.S.; Baumer, A.; Kaplan, V.; Albesa, M.; Sticht, H.; Rauch, A.; Puleo, C.; Hu, D. Identification of a novel loss-of-function calcium channel gene mutation in short QT syndrome (SQTS6). *Eur. Heart J.* **2011**, *32*, 1077–1088.
31. Sun, Y.; Zhang, P.; Li, X.; Guo, J. Inappropriate ICD discharge due to T-wave oversensing in a patient with short QT syndrome. *Pacing Clin. Electrophysiol.* **2010**, *33*, 113–116.
32. Villafane, J.; Young, M.L.; Maury, P.; Wolpert, C.; Anttonen, O.; Hamilton, R.; Kannankeril, P.J.; Fischbach, P.S. Short QT syndrome in a pediatric patient. *Pediatr. Cardiol.* **2009**, *30*, 846–850.
33. Rooryck, C.; Stef, M.; Burgelin, I.; Simon, D.; Souakri, N.; Thambo, J.B.; Chateil, J.F.; Lacombe, D.; Arveiler, B. 2.3 Mb terminal deletion in 12p13.33 associated with oculoauriculovertebral spectrum and evaluation of WNT5B as a candidate gene. *Eur. J. Med. Genet.* **2009**, *52*, 446–449.
34. Itoh, H.; Sakaguchi, T.; Ashihara, T.; Ding, W.G.; Nagaoka, I.; Oka, Y.; Nakazawa, Y.; Yao, T.; Jo, H.; Ito, M.; et al. A novel KCNH2 mutation as a modifier for short QT interval. *Int. J. Cardiol.* **2009**, *137*, 83–85.
35. Efremidis, M.; Letsas, K.P.; Weber, R.; Gavrielatos, G.; Filippatos, G.S.; Sideris, A.; Kardaras F. Recurrent syncope associated with a distinct ECG pattern consisting of short QT interval, early repolarization and atrioventricular block. Clin Res Cardiol. **2009**, *98*, 807–810.
36. Mizobuchi, M.; Enjoji, Y.; Yamamoto, R.; Ono, T.; Funatsu, A.; Kambayashi, D.; Kobayashi T.; Nakamura S. Nifekalant and disopyramide in a patient with short QT syndrome: Evaluation of pharmacological effects and electrophysiological properties. *Pacing Clin. Electrophysiol.* **2008**, *31*, 1229–1232.
37. Fichet, J.; Genee, O.; Pierre, B.; Babuty, D. Fatal QT interval. *Am. J. Emerg. Med.* **2008**, *26*, 739.e5-6.
38. Schimpf, R.; Bauersfeld, U.; Gaita, F.; Wolpert, C. Short QT syndrome: Successful prevention of sudden cardiac death in an adolescent by implantable cardioverter-defibrillator treatment for primary prophylaxis. *Heart Rhythm.* **2005**, *2*, 416–417.
39. Kirilmaz, A.; Ulusoy, R.E.; Kardesoglu, E.; Ozmen, N.; Demiralp, E. Short QT interval syndrome: A case report. *J. Electrocardiol.* **2005**, *38*, 371–374.
40. Hong, K.; Piper, D.R.; Diaz-Valdecantos, A.; Brugada, J.; Oliva, A.; Burashnikov, E.; Santos-de-Soto J.; Grueso-Montero J.; Diaz-Enfante E.; Brugada P.; et al. De novo KCNQ1 mutation responsible for atrial fibrillation and short QT syndrome in utero. *Cardiovasc. Res.* **2005**, *68*, 433–440.
41. Bellocq, C.; van Ginneken, A.C.; Bezzina, C.R.; Alders, M.; Escande, D.; Mannens, M.M.; Baró I.; Wilde A.A. Mutation in the KCNQ1 gene leading to the short QT-interval syndrome. *Circulation* **2004**, *109*, 2394–2397.
42. Zienciuk-Krajka, A.; Kukla, P.; Stec, S.; Raczak, G. Short QT syndrome presenting with atrial fibrillation and LV hypertrophy. *Int. J. Cardiol.* **2012**, *156*, e9.
43. van Schie, M.S.; Ramdat Misier, N.L.; van Leeuwen, W.J.; Taverne, Y.J.H.J.; de Groot, N.M.S. An unexpected finding by epicardial mapping: Atrial fibrillation in a 14-month-old patient with short QT syndrome. *Hear. Case Rep.* **2023**, *9*, 219–221.
44. Terlemez, S.; Çil, E.; Kula, S.; Oğuz, A.D.; Tunaoğlu, F.S. A diagnosis that escapes our attention: Short QT syndrome. *Gazi Med. J.* **2018**, *29*, 246–248.
45. Spartalis, M.; Livanis, E.; Spartalis, E.; Tsoutsinos, A. Electrical storm in an acquired short QT syndrome successfully treated with quinidine. *Clin. Case Rep.* **2019**, *7*, 1617–1618.
46. Portugal, G.; Martins Oliveira, M.; Silva Cunha, P.; Ferreira, F.; Lousinha, A.; Fiarresga, A.; Nogueira Da Silva M.; Cruz Ferreira R. Short QT syndrome presenting as syncope: How short is too short? *Rev. Port. De Cardiol.* **2014**, *33*, 649.e1–e6.
47. Morphet, J.A.M. The short QT syndrome and sudden infant death syndrome. *Can. J. Cardiol.* **2007**, *23*, 105.
48. Farag, M.J.; Atallah, J. Use of topical lidocaine in eliminating mechanically stimulated ventricular fibrillation in a patient with short QT syndrome. *Hear. Case Rep.* **2019**, *5*, 152–154.
49. Chevalier, P.; Moreau, A.; Richard, S.; Janin, A.; Millat, G.; Bessière, F.; Delinière A. Short QT interval as a harbinger of an arrhythmogenic cardiomyopathy. *Hear. Case Rep.* **2021**, *7*, 734–738.
