# Supplementary material for: The Uncommon Phenomenon of Short QT Syndrome: A Scoping Review of the Literature
Source: J Pers Med. 2025 Mar 8;15(3):105. doi: 10.3390/jpm15030105 (PMC11943495; doi:10.3390/jpm15030105)
Supplement: Supplementary file 1 [file jpm-15-00105-s001.zip › Supplementary Table S3 - Case series OK.pdf]

Supplementary Table S3. Table presenting available case series involving families or distinct cases with short QT syndrome.

Reference	Age	Gender	Presentation	Family history of SCD	ECG (QTc, ms)	Concomitant ECG changes	Genetic testing	Diagnostic investigations	Treatment/management	Comments
**Gussak et al, 2000 [1]**	**Family**									
	17y	F	Intraoperative AF	N/A	300 (Bazzett)	N	N/A	Chest x-ray, TTE, TEE, laboratory testing	Digoxin per os	Proband
	21y	M	Asymptomatic	N/A	267 (Bazzett)	N	N/A	N/A	N/A	Proband's brother
	57y	F	Palpitations	N/A	289 (Bazzett)	N	N/A	N/A	N/A	Proband's mother
	**Case**									
	37y	F	Syncopal episodes	N	248 (Bazzett)	N	N/A	N/A	N/A	SD awaiting scheduled diagnostic workup
**Sarquella – Brugada et al, 2015 [68]**	**Case 1**									
	Newborn (37w gestation)	F	AF since 24w gestational age	N/A	270	AF	p.V141M_ *KCNQ1* mutation	Chest x-ray, TTE, laboratory testing, 24h ECG Holter monitoring	PCM due to symptomatic pauses, CRT-D implantation, SCD at age 8y	Concomitant DCM
	**Case 2**									
	Newborn (37w gestation)	M	Fetal bradycardia since 26w gestational age, AF post-delivery	N/A	290	AF	p.V141M_ *KCNQ1* mutation	N/A	Oral quinidine, aspirin, unsuccessful electrical cardioversion, PCM	-
	**Case 3**									

	Newborn	F	Fetal bradycardia since 25w, AF post delivery	N/A	290	AF, junctional escape rhythm	p.V141M_ *KCNQ1* mutation	TTE, EPS	Aspirin, amiodarone, flecainide, successful EC, relapse at 4y	-
**Gaita et al, 2003 [60]**	**Family 1**									
	31y	F	Dizziness, palpitations	ϒ	<290 (Bazzett)	Left axis deviation	N/A	Exercise testing, 24h ECG Holter monitoring, HRV, TTE, CMR, EPS	ICD implantation	Proband
	35y	M	Syncope during exertion, PAF	ϒ	<280 (Bazzett)	N/A	N/A	Exercise testing, 24h ECG Holter monitoring, HRV, TTE, CMR, EPS	ICD implantation	Proband's brother
	6y	M	Syncope, palpitations, aborted CA	ϒ	<290 (Bazzett)	N/A	N/A	Exercise testing, 24h ECG Holter monitoring, HRV, TTE, CMR, EPS	N/A	Proband's child
	**Family 2**									
	67y	F	Palpitations, PAF	Y	295	N/A	N/A	Exercise testing, 24h ECG Holter monitoring, HRV, TTE, CMR, EPS	ICD implantation	Proband
	62y	F	AF	Y	250	N/A	N/A	Exercise testing, 24h ECG Holter monitoring, HRV, TTE, CMR, EPS	SD	Proband's sister
	15y	N	Syncope	Y	300	N/A	N/A	Exercise testing, 24h ECG Holter monitoring, HRV, TTE, CMR, EPS	ICD implantation	Proband's nephew
	40y	F	Asymptomatic	Y	268	N/A	N/A	Exercise testing, 24h ECG Holter	N/A	Proband's daughter

								monitoring, HRV, TTE, CMR, EPS		
**Priori et al, 2005 [67]**	**Family**									
	5y	F	Asymptomatic	N/A	315	Narrow peaked T-waves	G514A substitution in *KCNJ2*	24h ECG Holter monitoring, TTE, exercise test, laboratory testing	N/A	Proband
	35y	M	Presyncope, palpitations	N	320	N/A	G514A substitution in *KCNJ2*	24h ECG Holter monitoring, TTE, exercise test, laboratory testing, EPS	N/A	Proband's father
**Lu et al, 2006 [64]**	**Family**									
	30y	F	Palpitations, chest tightness, syncope, aborted CA (VF)	N	292 (Bazzett)	Peaked T-waves	N/A	TTE, laboratory testing, chest x-ray, 24h ECG Holter monitoring, brain CT	Metoprolol, amiodarone, magnesium per os<br><br>No EPS or ICD due to financial reasons	Proband
	7y	F	N/A	N/A	290	N/A	N/A	N/A	N/A	Proband's daughter
	53y	F	PAF	N/A	300	N/A	N/A	N/A	N/A	Proband's mother
**Bohora et al, 2009 [54]**	**Family**									
	27y	M	Dyspnea on exertion, palpitations	N	278	AF	Not performed	TTE, 24h ECG Holter monitoring, laboratory testing, exercise test	OAC, diuretics, ACE inhibitors	Proband<br>Concomitant DCM

	25y	M	Dyspnea, palpitations	N	302	AF, bradycardia	Not performed	TTE, 24h ECG Holter monitoring, laboratory testing, exercise test	OAC, diuretics, ACE inhibitors	Proband's brother Concomitant DCM
**Pirro et al, 2010 [66]**	**Family**									
	Newborn	F	N/A	Y	310	N/A	N588K mutation in *KCNH2*	24h ECG Holter monitoring	Oral hydroquinidine	Proband
	20y	M	Syncope, PAF	Y	N/A	N/A	N588K mutation in *KCNH2*	N/A	N/A	Proband's father
**Bun et al, 2012 [56]**	**Family**									
	28y	M	Aborted CA (VF)	N	320	N/A	Negative for known mutations	TTE, CMR, coronary angiography, laboratory testing	Oral hydroquinidine, ICD implantation	Proband
	N/A	F	N/A	N/A	N/A	N/A	Negative for known mutations	N/A	Oral hydroquinidine	Proband's mother
**Chinushi et al, 2012 [57]**	**Family**									
	38y	F	Syncope	Y	300	Peaked T-waves	N588K mutation in *KCNH2*	Exercise test, EPS	Denied ICD implantation and hydroquinidine, nifekalant challenge	Proband
	11y	M	Asymptomatic	Y	311	Peaked T-waves	N/A	N/A	N/A	Proband's son
**Ambrosini et al, 2014 [51]**	**Family**									

	9y	N/A	Epilepsy, intellectual disability, ASD	N/A	331	Narrow and peaked T-waves	c.1037A>C, p.K346T variant in *KCNJ2*	N/A	N/A	Twins
	9y	N/A	Epilepsy, intellectual disability, ASD	N/A	331	Narrow and peaked T-waves	c.1037A>C, p.K346T variant in *KCNJ2*	N/A	N/A	Twins
**Suzuki et al, 2014 [70]**	**Family**									
	10y	M	Asymptomatic	Y	260	N/A	N588K mutation in *KCNH2*	Laboratory testing, 24h ECG Holter monitoring, TTE, exercise testing	Oral Qquinidine, family denied ICD implantation	Proband
	N/A	F	N/A	Y	260	N/A	N588K mutation in *KCNH2*	N/A	N/A	Proband's mother
**Giustetto et al, 2015 [61]**	**Family**									
	16y	M	Asymptomatic	Y	300	Tall peaked T-waves in precordial leads, asymmetric negative T-waves in inferior leads	p.T6181 mutation in *KCNH2* (heterozygosis), p.K897T polymorphism in *KCNH2* (homozygosis)	TTE, CMR, 24h ECG Holter monitoring, exercise test, EPS	Oral hydroquinidine, sotalol per os, ILR	Proband
	34y	F	SCD	Y	300	N/A	N/A	N/A	N/A	Proband's mother
	21y	F	Asymptomatic	Y	340	N/A	p.T6181 mutation in *KCNH2* (heterozygosis), p.K897T	TTE, 24h ECG Holter monitoring, exercise test, EPS	Oral hydroquinidine, ILR	Proband's sister

							polymorphism in *KCNH2* (heterozygosis)			
	47y	F	Syncope	Y	344	Peaked T-waves in precordial leads	p.T6181 mutation in *KCNH2* (heterozygosis), p.K897T polymorphism in *KCNH2* (heterozygosis)	TTE, coronary angiogram, CMR	ICD implantation, oral hydroquinidine, sotalol per os	Maternal aunt
**Basarici et al, 2020 [53]**	**Family**									
	26y	F	Dizziness, atypical chest pain	Y	330	N/A	p.V141M mutation in *KCNQ1*	TTE, laboratory testing	ICD implantation after PCM extraction, sotalol per os	Proband<br>VVI PCM implantation due to SSS at 8y, upgrade at DDD at 21y following AF RFA
	N/A	M	N/A	N/A	N/A	N/A	N/A	N/A	N/A	Proband's father<br>VVI PCM implantation before death due to SSS. SQTS confirmed at ECG tracing
**Brugada et al, 2004 [55]**	**Family**									
	51y	M	Aborted CA	N/A	288	N/A	Negative for known mutations	EPS	ICD implantation	Proband
	20y	M	Asymptomatic	N/A	293	N/A	Negative for known mutations	N/A	N/A	Proband's son
**Hong et al, 2005 [63]**	**Family**									

	17y	F	AF and acute pulmonary oedema after cholecystectomy	N/A	225	AF	N588K mutation in *KCNH2*	EPS	Digoxin, propafenone, ICD implantation	Proband
	51y	F	Palpitations, PAF	N/A	230	AF	N588K mutation in *KCNH2*	EPS	ICD implantation, propafenone	Proband's mother
	84y	M	N/A	N/A	<300	AF, LVH	N588K mutation in *KCNH2*	N/A	N/A	Proband's grandfather
	21y	M	Asymptomatic	N/A	240	N/A	N588K mutation in *KCNH2*	EPS	ICD implantation, propafenone	Proband's brother
**Moriya et al, 2007 [65]**	**Case 1**									
	74y	F	N/A	N	333	N/A	N/A	Laboratory testing	Death due to IHD	Concomitant HF
	**Case 2**									
	26y	M	N/A	N	340	SSS	N/A	Laboratory testing	PCM implantation due to SSS	-
**Anttonen et al, 2008 [52]**	**Family**									
	18y	M	Aborted CA	N/A	<320	N/A	Negative for known mutations	N/A	N/A	Proband
	53y	M	Palpitations	N/A	<320	N/A	Negative for known mutations	Exercise testing, coronary angiography	N/A	Proband's father
	80y	M	Palpitations	N/A	<320	N/A	Negative for known mutations	N/A	N/A	Proband's grandfather

**Sun et al, 2011 [69]**	**Family**									
	45y	M	Dizziness	Y	298	Tall peaked T-waves in precordial leads	T6181 mutation in *KCNH2*	Laboratory testing, exercise testing, 24h ECG Holter monitoring, TTE, CMR, coronary angiography, EPS	ICD implantation	Proband
	N/A	F	N/A	Y	341	N/A	T6181 mutation in *KCNH2*	Laboratory testing, exercise testing, 24h ECG Holter monitoring, TTE, CMR, coronary angiography	Refused ICD implantation and medication	Proband's daughter
	N/A	F	N/A	Y	308	N/A	T6181 mutation in *KCNH2*	N/A	Refused ICD implantation and medication	Proband's daughter
	N/A	M	N/A	Y	315	N/A	T6181 mutation in *KCNH2*	N/A	Refused ICD implantation and medication	Proband's son
**Wisten et al, 2012 [71]**	**Family**									
	27y	F	Fatigue	Y	N/A	N/A	Negative for known mutations	N/A	Death at 27y	Proband (suspected)
	31y	M	Palpitations	N	260	N/A	Negative for known mutations	N/A	Death at 31y	Proband's brother
**Harrell et al, 2015 [62]**	**Case 1**									
	64y	M	Palpitations, near syncope	Y	319	PAF, atrial flutter,	c.1679T>C mutation in *KCNH2*	N/A	Refused ICD implantation	-

						peaked T-waves				
	**Family 1**									
	39Y	F	Aborted CA (VF)	Y	322	N/A	Mutation T618I c.1853C>T	N/A	ICD implantation, bepridil, bisoprolol per os	Proband
	N/A	M	N/A	Y	330	N/A	N/A	N/A	N/A	Proband's brother
	N/A	M	N/A	Y	330	N/A	N/A	N/A	N/A	Proband's nephew
	**Case 2**									
	10y	F	Fetal bradycardia, congenital SSS (22 weeks)	N/A	280	Bradycardia	c.421G>A in *KCNQ1*	N/A	PCM implantation	-
	**Family 2**									
	17y	F	Aborted CA (VF)	N/A	330	N/A	Negative for known mutations	N/A	ICD implantation	Proband
	N/A	F	N/A	N/A	320	N/A	N/A	N/A	N/A	Proband's grandmother
	N/A	F	N/A	Y	327	N/A	N/A	N/A	N/A	Proband's sister
	**Case 3**									
	42y	M	N/A	Y	340	Brugada–like ECG	Negative for known mutations	N/A	ICD implantation	-
**Thorsen et al, 2017 [5]**	**Case**									
	31y	M	Aborted CA (VF)	Y	320	N	*SLC4A3* c.1109 G>A variant (a total of 23 relatives in this	TTE	ICD implantation	Unexplained SD in 2 relatives at the age of 41 and 42y

							family were found carriers of the mutation)			
	**Family**									
	25y	M	SCD	N/A	320	N/A	*SLC4A3* c.1109 G>A variant Cascade screening revealed 2 more asymptomatic mutation-carrying relatives with short QTc in both	TTE	N/A	Proband's brother (Proband: SCD at 22y, no QTc available)
	61y	F	Asymptomatic	N/A	355	N/A	*SLC4A3* c.1109 G>A variant	TTE	Refused ICD implantation	Proband's mother
**Akdis et al, 2017 [50]**	**Case**									
	6mo	M	Syncope	Y	<320	N/A	c.1764C>G mutation in *KCNH2*, *KCNJ5* c.631C>T variant	EPS, TTE	ICD implantation	-
	**Family 1**									
	6y	F	Syncope	Y	320	N/A	c.1891T>G mutation in *KCNH2*, missense variant in	TTE	ICD implantation	Proband

							SCN10A c. 5605C>T			
	54y	M	Asymptomatic	Y	324	N/A	c.1891T>G mutation in *KCNH2*	TTE	Follow-up	Proband's father
	12y	F	Asymptomatic	Y	340	N/A	c.1891T>G mutation in *KCNH2*	TTE, CMR, exercise testing, 24h ECG Holter monitoring	Follow-up	Proband's sister Concomitant HFmrEF
	**Family 2**									
	17y	F	Aborted CA (VF)	Y	329	N/A	Loss of function *CACNA2D* mutation c.2264G>C, variant in dystrophin c.5010G>T (Trp1670Cys)	TTE	ICD implantation, b-blocker per os, lamotrigine	Proband Concomitant epilepsy
	45y	M	Asymptomatic	Y	357	N/A	Loss of function *CACNA2D* mutation c.2264G>C	Normal CV examinations	N/A	Proband's father
	N/A	F	Asymptomatic	N/A	N/A	N/A	Loss of function *CACNA2D* mutation c.2264G>C	Normal CV examinations	N/A	Proband's grandmother
**El Battrawy et al, 2018 [59]**	**Family**									

	29y	M	N/A	Y	257	N/A	p.N588K missense mutation in *KCNH2*	24h ECG Holter monitoring, TTE, exercise testing, CMR, EPS	ICD implantation	Proband
	N/A	F	N/A	Y	N/A (270 –300 ms)_	N/A	N/A	N/A	N/A	Proband's grandmother
	N/A	F	N/A	Y	N/A (270–300 ms)	N/A	N/A	N/A	N/A	Grandmother's sister
	N/A	F	N/A	Y	N/A (270–300 ms)	N/A	N/A	N/A	N/A	Proband's aunt
**El Battrawy et al, 2018 [58]**	**Family 1**									
	62y	M	N/A	Y	250	N/A	N/A	N/A	SCD	Proband
	N/A	F	Palpitations, syncope	Y	289	N/A	*KCNH2* mutation	TTE, CMR	ICD implantation, oral hydroquinidine	Proband's sister
	N/A	F	Palpitations, syncope	Y	287	N/A	*KCNH2* mutation	TTE, CMR	ICD implantation, oral hydroquinidine	Proband's niece
	N/A	M	Palpitations, syncope	Y	258	N/A	*KCNH2* mutation	TTE, CMR	ICD implantation, oral hydroquinidine	Proband's grandson
	**Family 2**									
	41y	M	AF	Y	347	Brugada-like ECG	G490R mutation in *CACNB2b*	EPS	ICD implantation, oral hydroquinidine	Proband
	N/A	F	N/A	Y	N/A	N/A	G490R mutation in *CACNB2b*	N/A	N/A	Proband's daughter

	N/A	F	N/A	Y	N/A	N/A	G490R mutation in *CACNB2b*	N/A	N/A	Proband’s daughter
	**Family 3**									
	25y	M	Aborted CA	N/A	330	ST elevation in lead V1	Mutation of *CACNB2b*	N/A	ICD implantation, oral hydroquinidine	Proband
	N/A	M	N/A	N/A	N/A	N/A	Mutation of *CACNB2b*	N/A	N/A	Proband’s brother
	N/A	F	N/A	N/A	N/A	N/A	Mutation of *CACNB2b*	N/A	N/A	Proband’s mother
	N/A	F	N/A	N/A	N/A	N/A	Mutation of *CACNB2b*	N/A	N/A	Proband’s aunt
	N/A	F	N/A	N/A	N/A	N/A	Mutation of *CACNB2b*	N/A	N/A	Proband’s aunt
	N/A	M	N/A	N/A	N/A	N/A	Mutation of *CACNB2b*	N/A	N/A	Proband’s cousin
	**Case 1**									
	67y	F	Palpitations	Y	320	N/A	N/A	N/A	N/A	4 other cases of SCD in the family
	**Case 2**									
	62y	M	Palpitations, recurrent syncope	N/A	272	N/A	N/A	EPS	Refused ICD implantation and treatment with oral hydroquinidine	-
	**Case 3**									
	34y	F	Recurrent syncope	N/A	320	N/A	N/A	TTE, 24h ECG Holter monitoring, CMR	ILR implantation	-
	**Case 4**									
	28y	M	Recurrent palpitations	N	307	AF, atrial flutter	N/A	N/A	Oral hydroquinidine	-

**Giustetto et al, 2007 [74]**	**Family**									
	20y	M	Syncope	Y	260	• Sinus bradycardia<br>• Left axis deviation<br>• Tall/peaked T-waves<br>• Absent ST interval	N/A	Laboratory testing, TTE, exercise testing, EPS	Flecainide per os, ICD implantation	Proband<br>3 other cases of SCD in the family
	N/A	F	Dizziness, palpitations	Y	280	N/A	N/A	EPS	ICD implantation	Proband's sister
**Gimeno et al, 2011 [75]**	**Family**									
	36y	M	Palpitations	N/A	342-385	• Sinus bradycardia<br>• ERP<br>• Peaked T-waves	K897T mutation in *KCNH2*, S38G mutation in *KCNE1*	24h ECG Holter monitoring, TTE, procainamide drug challenge	Follow-up	Proband
	41y	F	N/A	N/A	365	None	N/A	N/A	N/A	Proband's sister<br>History of SVT and PAF
	39y	M	N/A	N/A	350	N/A	N/A	N/A	N/A	Proband's brother
**Babaoğlu et al, 2012 [76]**	**Family**									
	13y	M	Chest pain	Y	300	• Narrow, symmetric, tall T-waves	N/A	Chest x-ray, TTE, laboratory testing, 24h ECG Holter monitoring,	N/A	Proband

						• Absent ST-segment		exercise testing, EPS		
	N/A	F	Asymptomatic	Y	320	• Narrow, symmetric, tall T-waves<br>• Absent ST-segment	N/A	N/A	N/A	Proband's mother
**Schneider et al, 2021 [72]**	**Family**									
	10y	F	Aborted CA due to VF	Y	344	N/A	3 VUS	Laboratory testing, TTE, CMR, exercise testing	ICD implantation	Proband<br>First reported case of *KCNQ1* mutation
	N/A	F	N/A	Y	327	N/A	VUS	N/A	N/A	Proband's mother carrying the same mutation
**Peters et al, 2011 [73]**	**Cases**									
	36y	M	Syncope, chest pain	N	340	• Prolonged QRS<br>• ST-elevation in lead V2<br>• Small epsilon wave in V2	Not performed	TTE, coronary angiography, ajmaline drug testing	Follow-up	Dilation of RVOT in TTE, right precordial ST-segment elevation after ajmaline testing
	29y	M	Syncope, known epilepsy	N	340	• U-waves in V2, V3	P2006A mutation in *SCN5A*	TTE, EPS, ajmaline drug testing	Follow-up	Apical akinesia of the RV with prominent moderator band, right precordial ST-

						• Right precordial QRS prolongation				segment elevation after ajmaline testing
	54y	F	Syncope	N	360	• T-wave inversion in leads V1, V2	Negative for known mutations	Coronary angiography, TTE, EPS, 24h ECG Holter monitoring, ajmaline drug testing	ICD implantation, ICD explantation 4 years later after a series of negative EPSs	Apical and inferior akinesia of the RV, right precordial ST-segment elevation after ajmaline testing

Abbreviations: ACE, angiotensin-converting enzyme; AF, atrial fibrillation; ASD, autism spectrum disease; CA, cardiac arrest; CMR, cardiovascular magnetic resonance; CRT-D, cardiac resynchronization therapy–defibrillator; CT, computed tomography; CV, cardiovascular; DCM, dilative cardiomyopathy; EC, electrical cardioversion; ECG, electrocardiogram; EPS, electrophysiological study; HF, heart failure; HFmrEF, heart failure with mildly reduced ejection fraction; HRV, heart rate variability; ICD, implantable cardioverter defibrillator; IHD, ischemic heart disease; ILR, implantable loop recorder; LVH, left ventricular hypertrophy; OAC, oral anticoagulation; PAF, paroxysmal atrial fibrillation; PCM, pacemaker; PCD, primary carnitine deficiency; RFA, radiofrequency ablation; RV, right ventricle; RVOT, right ventricular outflow tract; SCD, sudden cardiac death; SD, sudden death; SSS, sick sinus syndrome; SVT, supraventricular tachycardia; TEE, transesophageal echocardiogram; VUS, variant of unknown significance; TTE, transthoracic echocardiogram; VF, ventricular fibrillation.

References

1. Gussak, I.; Brugada, P.; Brugada, J.; Wright, R.S.; Kopecky, S.L.; Chaitman, B.R.; Bjerregaard, P. Idiopathic short QT interval: A new clinical syndrome? *Cardiology* **2000**, *94*, 99–102.

5. Thorsen, K.; Dam, V.S.; Kjaer-Sorensen, K.; Pedersen, L.N.; Skeberdis, V.A.; Jurevičius, J.; Treinys, R.; Petersen, I.M.B.S.; Nielsen, M.S.; Oxvig, C.; et al. Loss-of-activity-mutation in the cardiac chloride-bicarbonate exchanger AE3 causes short QT syndrome. *Nat. Commun.* **2017**, *8*, 1696.

50. Akdis, D.; Saguner, A.M.; Medeiros-Domingo, A.; Schaller, A.; Balmer, C.; Steffel, J.; Brunckhorst C.; Duru F. Multiple clinical profiles of families with the short QT syndrome. *Europace* **2018**, *20*, f113–f121.

51. Ambrosini, E.; Sicca, F.; Brignone, M.S.; D'Adamo, M.C.; Napolitano, C.; Servettini, I.; Moro F.; Ruan Y.; Guglielmi L.; Pieroni S.; et al. Genetically induced dysfunctions of Kir2.1 channels: Implications for short QT3 syndrome and autism-epilepsy phenotype. *Hum. Mol. Genet.* **2014**, *23*, 4875–4886.

52. Anttonen, O.; Väänänen, H.; Junttila, J.; Huikuri, H.V.; Viitasalo, M. Electrocardiographic transmural dispersion of repolarization in patients with inherited short QT syndrome. *Ann. Noninvasive. Electrocardiol*. **2008**, *13*, 295–300.

53. Basarici, I. Delayed diagnosis of short QT syndrome concealed by pacemaker implant due to sick sinus syndrome. *Anatol. J. Cardiol.* **2020**, *23*, 111–113.

54. Bohora, S.; Namboodiri, N.; Tharakan, J.; Vk, A.K.; Nayyar, S. Dilated cardiomyopathy with short QT interval: Is it a new clinical entity? *Pacing Clin. Electrophysiol.* **2009**, *32*, 688–690.

55. Brugada, R.; Hong, K.; Dumaine, R.; Cordeiro, J.; Gaita, F.; Borggrefe, M.; Menendez T.M.; Brugada J.; Pollevick G.D.; Wolpert C.; et al. Sudden death associated with short-QT syndrome linked to mutations in HERG. *Circulation* **2004**, *109*, 30–35.

56. Bun, S.S.; Maury, P.; Giustetto, C.; Deharo, J.C. Electrical storm in short-QT syndrome successfully treated with Isoproterenol. *J. Cardiovasc. Electrophysiol.* **2012**, *23*, 1028–1030.

57. Chinushi, M.; Sato, A.; Izumi, D.; Furushima, H. Nifekalant enlarged the transmural activation-recovery interval difference as well as the peak-to-end interval on surface ECG in a patient with short-QT syndrome. *J. Cardiovasc. Electrophysiol.* **2012**, *23*, 877–880.

58. El-Battrawy, I.; Besler, J.; Liebe, V.; Schimpf, R.; Tülümen, E.; Rudic, B.; Lang S.; Wolpert C.; Zhou X.; Akin I.; et al. Long-Term Follow-Up of Patients With Short QT Syndrome: Clinical Profile and Outcome. *J. Am. Heart Assoc.* **2018**, *7*, e010073.

59. El-Battrawy, I.; Lan, H.; Cyganek, L.; Zhao, Z.; Li, X.; Buljubasic, F.; Lang S.; Yücel G.; Sattler K.; Zimmermann W.H.; et al. Modeling Short QT Syndrome Using Human-Induced Pluripotent Stem Cell-Derived Cardiomyocytes. *J. Am. Heart Assoc.* **2018**, *7*, e007394.

60. Gaita, F.; Giustetto, C.; Bianchi, F.; Wolpert, C.; Schimpf, R.; Riccardi, R.; Grossi S.; Richiardi E.; Borggrefe M. Short QT Syndrome: A familial cause of sudden death. *Circulation* **2003**, *108*, 965–970.

61. Giustetto, C.; Scrocco, C.; Giachino, D.; Rapezzi, C.; Mognetti, B.; Gaita, F. The lack of effect of sotalol in short QT syndrome patients carrying the T618I mutation in the KCNH2 gene. *Hear. Case Rep.* **2015**, *1*, 373–378.

62. Harrell, D.T.; Ashihara, T.; Ishikawa, T.; Tominaga, I.; Mazzanti, A.; Takahashi, K.; Oginosawa Y.; Abe H.; Maemura K.; Sumitomo N.; et al. Genotype-dependent differences in age of manifestation and arrhythmia complications in short QT syndrome. *Int. J. Cardiol.* **2015**, *190*, 393–402.
63. Hong, K.; Bjerregaard, P.; Gussak, I.; Brugada, R. Short QT syndrome and atrial fibrillation caused by mutation in KCNH2. *J. Cardiovasc. Electrophysiol.* **2005**, *16*, 394–396.
64. Lu, L.X.; Zhou, W.; Zhang, X.; Cao, Q.; Yu, K.; Zhu, C. Short QT syndrome: A case report and review of literature. *Resuscitation* **2006**, *71*, 115–121.
65. Moriya, M.; Seto, S.; Yano, K.; Akahoshi, M. Two cases of short QT interval. *Pacing Clin. Electrophysiol.* **2007**, *30*, 1522–1526.
66. Pirro, E.; De Francia, S.; Banaudi, E.; Riggi, C.; De Martino, F.; Piccione, F.M.; Giustetto C.; Racca S.; Agnoletti G.; Di Carlo F. Short QT syndrome in infancy. Therapeutic drug monitoring of hydroquinidine in a newborn infant. *Br. J. Clin. Pharmacol.* **2011**, *72*, 982–984.
67. Priori, S.G.; Pandit, S.V.; Rivolta, I.; Berenfeld, O.; Ronchetti, E.; Dhamoon, A.; Napolitano C.; Anumonwo J.; di Barletta M.R.; Gudapakkam S.; et al. A novel form of short QT syndrome (SQT3) is caused by a mutation in the KCNJ2 gene. *Circ. Res.* **2005**, *96*, 800–807.
68. Sarquella-Brugada, G.; Campuzano, O.; Iglesias, A.; Grueso, J.; Bradley, D.J.; Kerst, G.; Shmorhun D.; Brugada J.; Brugada R. Short QT and atrial fibrillation: A KCNQ1 mutation-specific disease. Late follow-up in three unrelated children. *Hear. Case Rep.* **2015**, *1*, 193–197.
69. Sun, Y.; Quan, X.Q.; Fromme, S.; Cox, R.H.; Zhang, P.; Zhang, L.; Guo D.; Guo J.; Patel C.; Kowey P.R.; et al. A novel mutation in the KCNH2 gene associated with short QT syndrome. *J. Mol. Cell Cardiol.* **2011**, *50*, 433–441.
70. Suzuki, H.; Hoshina, S.; Ozawa, J.; Sato, A.; Minamino, T.; Aizawa, Y.; Saitoh A. Short QT syndrome in a boy diagnosed on screening for heart disease. *Pediatr. Int.* **2014**, *56*, 774–776.
71. Wisten, A.; Boström, I.M.; Mörner, S.; Stattin, E.L. Mutation analysis of cases of sudden unexplained death, 15 years after death: Prompt genetic evaluation after resuscitation can save future lives. *Resuscitation* **2012**, *83*, 1229–1234.
72. Schneider, K.; Parrott, A.; Spar, D.; Knilans, T.; Czosek, R.; Miller, E.; Anderson J. A novel variant in KCNQ1 associated with short QT syndrome. *Hear. Case Reports.* **2021**, *7*, 650–654.
73. Peters, S.; Trümmel, M.; Koehler, B. Shorter-than-normal QT interval and provocable right precordial ST segment elevation in three patients with suspicious arrhythmogenic right ventricular cardiomyopathy. *J. Fur Kardiol.* **2011**, *18*, 326–328.
74. Giustetto, C.; Gaita, F. Syncope in a Patient with a Short QT Interval. *Syncope Cases* **2007**, 177–179. Doi: 10.1002/9780470995013.ch62
75. Gimeno, J.R.; Lacunza, J.; García-Molina, E.; Oliva-Sandoval, M.J.; Valdes, M. Short QT and dilated cardiomyopathy. A phenotype with a good prognosis? *Int. J. Cardiol.* **2011**, *151*, 356–357.
76. Babaoğlu, K.; Binnetoğlu, K.; Altun, G.; Tuzcu, V. A 13-year-old boy with a short QT interval—Case report. *Anatol. J. Cardiol.* **2012**, *12*, 275.
