# Supplementary material for: The Uncommon Phenomenon of Short QT Syndrome: A Scoping Review of the Literature
Source: J Pers Med. 2025 Mar 8;15(3):105. doi: 10.3390/jpm15030105 (PMC11943495; doi:10.3390/jpm15030105)
Supplement: Supplementary file 1 [file jpm-15-00105-s001.zip › Supplementary Table S4 OK.pdf]

Supplementary Table S4. Studies assessing healthy populations or health databases for the presence of short QT syndrome.

STUDY ID	PATIENTS	STUDY ASSESSMENTS/INTERVENTIONS	COMPARISONS	OUTCOMES
**Makarov et al, 2006 [130]**	1,531 healthy children and adolescents	ECG analysis	-	• QTc<350 ms was registered in 12 children<br>• 8 of these children (mean QTc 329.1±32.3 ms) had family history of syncope or SD
**Gallagher et al, 2006 [135]**	12,012 healthy subjects undergoing routine medical examinations	ECG analysis	-	• The shortest QTc was 335 ms<br>• QTc≤330 ms is extremely rare in healthy subjects<br>• The presence of a QT interval in the lowest 1/2 centile of the normal range does not imply a significant risk of SD
**Anttonen et al, 2007 [123]**	882 randomly selected subjects<br>• Median age: 44±8.4y<br>• Follow-up for 29±10y	• Follow-up for all-cause and CV mortality<br>• QTc assessment (Bazzett, Fridericia and nomogram formulas)	-	• The prevalence of QT<320 ms based on QTc, QTfc, and QTnc was 0.10%, 0.08%, and 0.06%, and the prevalence of QT<340 ms was 0.4%, 0.3%, and 0.3%, respectively<br>• The majority of subjects with SQT were males<br>• All-cause or CV mortality did not differ between subjects with a very short or SQT and those with normal QT (360-450 ms)<br>• There were no SCD, aborted SCD, or documented ventricular tachyarrhythmias among subjects with QTfc <340 ms<br>• A SQT does not indicate an increased risk for all-cause or CV mortality in middle-aged, community-based patients
**Moriya et al, 2007 [65]**	19,153 subjects undergoing health examination in Japan<br>• Follow-up for 26.38±14.83y	• ECG analysis<br>• Laboratory testing	-	2 subjects met the criteria of SQT interval of <350 ms (prevalence: 0.01%). The 2 identified SQT cases are presented in case series table (Moriya et al) (65)
**Funada et al, 2008 [114]**	10,984 subjects who received a consultation at Kanazawa University Hospital, Japan	ECG analysis	-	• QTc values were significantly longer in females than in males<br>• Among 5,511 males, 69 (1.25%) exhibited QTc<354 ms (2 SDs below the mean in males), and among 5,473 females, 89 (1.63%) exhibited QTc < 364 ms (2 SDs below the mean in females)<br>• Only 3 subjects (0.03% in all subjects and 0.05% in males) had QTc<300 ms, however, none had clinical symptoms of SQTS
**Kobza et al, 2009 [133]**	41,767 Swiss citizens	ECG analysis	-	• The mean QTc was 394±22 ms<br>• 1% of the conscripts had a QTc<347ms<br>• None of the subjects presented a QTc<300 ms<br>• The prevalence of QTc<320 ms was 0.02%
**Lubart et al, 2009 [99]**	422 patients hospitalized in an acute geriatric care ward	ECG analysis (file screening)	-	• SQT was found in 30 patients (7.1%) in lead II and in 19 (4.5%) by the mean of 12 leads<br>• SQT was related to a higher HR, chronic AF and schizophrenia
**Lubart et al, 2012 [132]**	178 patients from a long-term geriatric care ward	ECG analysis	-	• SQT was found in 7 residents (4%) and was not related to any parameter
**Miyamoto et al, 2012 [94]**	114,334 patients from a database	ECG analysis	-	• 427 patients (mean age 43.4±22.4 years) had a SQT (234 men)<br>• QTc was significantly longer in females than in males (363.8±6.1 ms vs 357.1±5.8 ms, P<0.0001)

				• AF and ERP were complicated with SQT in 39 (9.1%) and 26 (6.1%) patients, respectively<br>• During the follow-up, 2 patients, 1 of whom had ERP, developed life-threatening events, in contrast to 6 patients who died of noncardiac causes and did not have ERP
**Panicker et al, 2012 [92]**	1,886 healthy subjects	ECG analysis	-	4 subjects in each group (with and without early repolarization) had SQT (QTc ⩽ 350 ms), hence a total of 8 subjects in the study
**Iribarren et al, 2014 [106]**	Database of 6.4 million ECGs obtained between 1995-2008 among 1.7 million persons	• ECG analysis<br>• Internal, population-based method for HR correction (QTcreg)<br>• ECGs with QTcreg ≤300 ms were manually validated	-	• Out of the total ECGs analyzed, 1,086 had a machine-read QTcreg≤300 ms<br>• Only 4% (45/1,086) were validated yielding a prevalence of 0.7 per 100,000 or 1 of 141,935 ECGs<br>• The overall prevalence of QTcreg≤300 ms was 2.7 per 100,000 or 1 of 37,335<br>• Age >65 years, black race, prior history of ventricular arrhythmias, COPD, ST-T abnormalities, myocardial ischemia, bigeminy pattern, and digitalis effect were significantly associated with validated QTcreg≤300 ms<br>• Validated QTcreg≤300 ms was associated with a 2.6-fold increased risk of death within 8.3 years of follow-up
**Pickham et al, 2014 [90]**	1,417 U.S. athletes undergoing pre-participation cardiovascular screening	ECG analysis	-	• 7.8% of athletes (n=110) had an abnormal variant according to the European criterion (QTc<380 ms)<br>• No athletes were below thresholds according to the Stanford (QTc<340 ms) and Seattle (QTc<320 ms) criteria
**Guerrier et al, 2015 [108]**	272,504 ECGs of 99,380 patients (age <21y)	• Retrospective ECG analysis<br>• Electronically measured QTc of 140-340 ms confirmed by a pediatric electrophysiologist and identified for chart review for associated clinical characteristics, symptoms, and outcomes	-	• 45 patients (76% male) had QTc≤340 ms, (0.05% prevalence) with a median age of 15 (2-17) years, median QT of 330 (280-360) ms, and median QTc of 323 (313-332) ms<br>• Women had significantly shorter QTc compared to men
**Dhutia et al, 2016 [120]**	18,825 apparently healthy people aged 14-35y	CV evaluation (history, physical examination, ECG)	-	• The prevalence of SQT was 0.1% (26 patients, QTc≤320 ms), 0.2% (44 patients, QTc≤330 ms), 7.9% (1,478 patients, QTc<380 ms), 15.8% (2,973 patients, QTc<390 ms)<br>• Male gender and Afro-Caribbean ethnicity had the strongest association with SQT<br>• Athletes had shorter QT intervals than non-athletes<br>• Individuals with SQT≤320 ms did not report syncope or a family history of SD
**Kaiser – Nielsen et al, 2017 [103]**	201 athletes referred for cardiac evaluation at a Sports Cardiology Clinic in Denmark (13-66y)	• Personal and family history<br>• ECG analysis<br>• TTE	-	SQT was found in 1 athlete (QTc<320 ms)
**Hazeki et al, 2018 [134]**	75,040 ECGs of participants in a school-based screening program in Japan	ECG analysis	-	The prevalence of SQTS was estimated at 1/30,000–1/10,000
**Rabkin et al, 2020 [88]**	Hospital ECG database	ECG analysis (screening for cases with a QTc<340 ms, Bazett's formula)	Age- and sex-matched cohort obtained from	• QTc was recalculated using the spline (QTcRBK) formula, which more accurately adjusts for the HR and

			individuals with normal QT intervals	identifies cases based on percentile distribution of the QT interval<br>• There were 28 cases with a SQTc (QTcRBK<380 ms) with a mean age of 69.6±14.6 years (50% male)<br>• QTc was 305.7±61.1 ms with Bazzett's formula and with QTcRBK: 308.4±31.4 ms<br>• Subsequent ECGs showed atrial flutter in 21%, AF in 18%, and AT in 4% of cases, an incidence of AAs significantly greater than in age- and sex-matched controls
**Nikoo et al, 2022 [93]**	4,363 adult subjects from Kherameh cohort study	• ECG analysis<br>• ECGs with QTc < 370 ms were reanalyzed for bradycardia, ERP, AF and other conduction abnormalities	-	• 72 subjects (1.65%) had QTc<370 ms (mean QTc: 360.72±11.72), with male predominance and lower HR compared to subjects with normal QTc<br>• At least 2 subjects with high-probability for SQTS and 3 with intermediate-probability for SQTS were identified<br>• The incidence of cardiac symptoms/events, familial SCDs and ECG-derived specific findings were high amongst SQTS-susceptible index persons
**Adler et al, 2016 [125]**	720 patients referred to a specialized inherited arrhythmia clinic	Follow-up for outcomes for a median of 4.1y	-	• In diagnosed channelopathy or ARVC, 44 patients received an ICD for secondary prevention (LQTS, 9; BrS, 8; CPVT, 3; SQTS, 1; and ARVC, 23)<br>• In individuals determined to have clinical or genetic disease by cascade screening, no SCD occurred over a median follow-up of 5.6 years<br>• Low event rates occurred despite a low rate of ICD implantation for primary prevention
**Providencia et al, 2018 [89]**	14,662 young adults from the SCD-SOS cohort	QTc estimation using 4 different formulae	-	• The prevalence of QTc≤330 and ≤320 ms was extremely low (≤0.07% and≤0.02%, respectively), more frequently identified by the Framingham formula<br>• The different QTc correction formulae led to a shift in SQTS probability in 5%-10% of individuals<br>• Intermediate probability individuals were rare (<0.1%), and no high-SQTS probability individuals were identified
**Conte et al, 2024 [140]**	522 patients with IAS and AAs from 28 centers in 16 countries	International retrospective registry – screening for IAS with concomitant AAs	-	• 522 patients with IAS and AAs were identified<br>• Most patients were diagnosed with Brugada syndrome and LQTS (68% and 18% respectively)<br>• The remaining 14% presented with SQTS, ERP syndrome, CPVT, progressive cardiac conduction diseases and IVF<br>• SQTS is the IAS with the higher risk for AF (30%)
**Ahmadi-Renani et al, 2024 [139]**	7,678 ECGs from the TeCS database, from individuals aged >35 years	Demographics, clinical characteristics and ECG analysis	-	SQT (QTc <330 ms in men and <340 ms in women) was found in 0.03% of participants, which corresponds in only 3 cases

Abbreviations: AA, atrial arrhythmia; AAS, anabolic androgenic steroids; AF, atrial fibrillation; ARVC, arrhythmogenic right ventricular cardiomyopathy; AT, atrial tachycardia; COPD, chronic obstructive pulmonary disease; CV, cardiovascular; CVPT, catecholaminergic polymorphic tachycardia; DCM, dilated cardiomyopathy; ECG, electrocardiogram; ERP, early repolarization; HR, heart rate; IAS, inherited arrhythmia syndrome; IVF, idiopathic ventricular fibrillation; LQTS, long QT syndrome; SCD, sudden cardiac death; SD, standard deviation; SQTS, short QT syndrome; SQT, short QT; TTE, transthoracic echocardiogram.

References

65. Moriya, M.; Seto, S.; Yano, K.; Akahoshi, M. Two cases of short QT interval. *Pacing Clin. Electrophysiol.* **2007**, *30*, 1522–1526.

88. Rabkin, S.W.; Tang, J.K.K. The Short QTc Is a Marker for the Development of Atrial Flutter and Atrial Fibrillation. *Cardiol. Res. Pract.* **2020**, *2020*, 2858149.
89. Providência, R.; Karim, N.; Srinivasan, N.; Honarbakhsh, S.; Vidigal Ferreira, M.J.; Gonçalves, L.; Marijon E.; Lambiase P.D. Impact of QTc formulae in the prevalence of short corrected QT interval and impact on probability and diagnosis of short QT syndrome. *Heart* **2018**, *104*, 502–508.
90. Pickham, D.; Zarafshar, S.; Sani, D.; Kumar, N.; Froelicher, V. Comparison of three ECG criteria for athlete pre-participation screening. *J. Electrocardiol.* **2014**, *47*, 769–774.
92. Panicker, G.K.; Manohar, D.; Karnad, D.R.; Salvi, V.; Kothari, S.; Lokhandwala, Y. Early repolarization and short QT interval in healthy subjects. *Heart Rhythm.* **2012**, *9*, 1265–1271.
93. Nikoo, M.H.; Heiran, A.; Mashayekh, F.; Rezaianzadeh, A.; Shiravani, A.; Azadian, F. A descriptive report on short QT interval in Kherameh branch of the PERSIAN cohort study. *Sci. Rep.* **2022**, *12*, 2898.
94. Miyamoto, A.; Hayashi, H.; Yoshino, T.; Kawaguchi, T.; Taniguchi, A.; Itoh, H.; Sugimoto Y.; Itoh M.; Makiyama T.; Xue J.Q.; et al. Clinical and electrocardiographic characteristics of patients with short QT interval in a large hospital-based population. *Heart Rhythm.* **2012**, *9*, 66–74.
99. Lubart, E.; Segal, R.; Yearovoi, A.; Fridenson, A.; Baumoehl, Y.; Leibovitz, A. QT interval disturbances in hospitalized elderly patients. *Isr. Med. Assoc. J.* **2009**, *11*, 147–150.
103. Kaiser-Nielsen, L.V.; Tischer, S.G.; Prescott, E.B.; Rasmusen, H.K. Symptoms, diagnoses, and sporting consequences among athletes referred to a Danish sports cardiology clinic. *Scand. J. Med. Sci. Sports.* **2017**, *27*, 115–123.
106. Iribarren, C.; Round, A.D.; Peng, J.A.; Lu, M.; Klatsky, A.L.; Zaroff, J.G.; Holve T.J.; Prasad A.; Stang P. Short QT in a cohort of 1.7 million persons: Prevalence, correlates, and prognosis. *Ann. Noninvasive. Electrocardiol.* **2014**, *19*, 490–500.
108. Guerrier, K.; Kwiatkowski, D.; Czosek, R.J.; Spar, D.S.; Anderson, J.B.; Knilans, T.K. Short QT Interval Prevalence and Clinical Outcomes in a Pediatric Population. *Circ. Arrhythm. Electrophysiol.* **2015**, *8*, 1460–1464.
114. Funada, A.; Hayashi, K.; Ino, H.; Fujino, N.; Uchiyama, K.; Sakata, K.; Masuta E.; Sakamoto Y.; Tsubokawa T.; Yamagishi M. Assessment of QT intervals and prevalence of short QT syndrome in Japan. *Clin. Cardiol.* **2008**, *31*, 270–274.
120. Dhutia, H.; Malhotra, A.; Parpia, S.; Gabus, V.; Finocchiaro, G.; Mellor, G.; Merghani A.; Millar L.; Narain R.; Sheikh N.; et al. The prevalence and significance of a short QT interval in 18,825 low-risk individuals including athletes. *Br. J. Sports Med.* **2016**, *50*, 124–129.
123. Anttonen, O.; Junttila, M.J.; Rissanen, H.; Reunanen, A.; Viitasalo, M.; Huikuri, H.V. Prevalence and prognostic significance of short QT interval in a middle-aged Finnish population. *Circulation* **2007**, *116*, 714–720.
125. Adler, A.; Sadek, M.M.; Chan, A.Y.; Dell, E.; Rutberg, J.; Davis, D.; Green M.S.; Spears D.A.; Gollob M.H. Patient Outcomes From a Specialized Inherited Arrhythmia Clinic. *Circ. Arrhythm. Electrophysiol.* **2016**, *9*, e003440.
130. Makarov, L.M.; Kisileva, I.I.; Dolgikh, V.V.; Bimbaev, A.B.Z.; Bairova, T.A.; Drozdova, A.I. Assesment of parameters of QT interval in children and adolescents. *Kardiologiya* **2006**, *46*, 37–41.
132. Lubart, E.; Segal, R.; Megid, S.; Yarovoy, A.; Leibovitz, A. QT interval disturbances in elderly residents of long-term care facilities. *Isr. Med. Assoc. J.* **2012**, *14*, 244–246.
133. Kobza, R.; Roos, M.; Niggli, B.; Abächerli, R.; Lupi, G.A.; Frey, F.; Schmid J.J.; Erne P. Prevalence of long and short QT in a young population of 41,767 predominantly male Swiss conscripts. *Heart Rhythm.* **2009**, *6*, 652–657.
134. Hazeki, D.; Ninomiya, Y.; Ueno, K.; Yoshinaga, M. Tentative screening criteria for short QT interval in children and adolescents. *Circ. J.* **2018**, *82*, 2627–2633.
135. Gallagher, M.M.; Magliano, G.; Yap, Y.G.; Padula, M.; Morgia, V.; Postorino, C.; Liberato F.; Leo R.; Borzi M.; Romeo F. Distribution and Prognostic Significance of QT Intervals in the Lowest Half Centile in 12,012 Apparently Healthy Persons. *Am. J. Cardiol.* **2006**, *98*, 933–935.
139. Ahmadi-Renani, S.; Soltani, D.; Farshbafnadi, M.; Shafiee, A.; Jalali, A.; Mohammadi, M.; Golestanian S.; Kamalian E.; Alaeddini F.; Saadat S.; et al. Prevalence and associated factors of ECG abnormality patterns indicative of cardiac channelopathies among adult general population of Tehran, Iran: A report from the Tehran Cohort Study (TeCS). *BMC Cardiovasc. Disord.* **2024**, *24*, 566.
140. Conte, G.; Bergonti, M.; Probst, V.; Morita, H.; Tfelt-Hansen, J.; Behr, E.R.; Kengo K.; Arbelo E.; Crotti L.; Sarquella-Brugada G.; et al. aTrial arrhythmias in inhEriTed aRrhythmIa Syndromes: Results from the TETRIS study. *Europace* **2024**, *26*, euae 288. DOI: 10.1093/europace/euae288
