# Supplementary material for: The Uncommon Phenomenon of Short QT Syndrome: A Scoping Review of the Literature
Source: J Pers Med. 2025 Mar 8;15(3):105. doi: 10.3390/jpm15030105 (PMC11943495; doi:10.3390/jpm15030105)
Supplement: Supplementary file 1 [file jpm-15-00105-s001.zip › Supplementary Table S5 OK.pdf]

Supplementary Table S5. Studies implementing genetic testing in patients with short QT syndrome.

STUDY ID	PATIENTS	STUDY ASSESSMENTS/INTERVENTIONS	OUTCOMES
**Brugada et al, 2004 [55]**	3 families with SQTS (9 patients in total) with IDs: 30-335, 30-339, 30-371	• Genetic analysis<br>• In vitro transcription and mammalian cell transfection<br>• EPS	• Same family described in case series by Gaita et al (60)<br>• Missense mutation (C to G substitution at nucleotide 1,764) identified in family 30-371 in *KCNH2*<br>• Analysis of family 30-335 identified a different missense mutation in the same residue (C to A substitution at nucleotide 1,764) in *KCNH2*<br>• Both mutations substituted the asparagine at codon 588 in *KCNH2* protein (HERG)<br>• No mutations in family 30-339
**Antzelevitch et al, 2007 [137]**	82 patients with BrS	Genetic screening	• 3 probands with ST-segment elevation and QTc≤360 ms had mutations in genes encoding the cardiac L-type calcium channel<br>• QTc ranged from 330-370 ms among probands and clinically affected family members<br>• Quinidine normalized QT and prevented stimulation-induced VT<br>• Genetic and heterologous expression studies revealed loss-of-function missense mutations in *CACNA1C* (A39V and G490R) and *CACNB2* (S481L) encoding the α1- and β2b-subunits of the L-type calcium channel<br>• Confocal microscopy revealed a defect in trafficking of A39V Cav1.2 channels but normal trafficking of channels containing G490R Cav1.2 or S481L Cavβ2b-subunits
**Burashnikov et al, 2010 [136]**	205 patients with inherited channelopathies<br>• 162 patients with BrS and BrS+SQTS<br>• 19 patients with IVF<br>• 24 patients with ERS	Genetic screening	• 23 mutations were identified in total<br>• 16% of BrS/BrS+SQT, IVF, and ERS probands displayed mutations in α1, β2, and α2δ subunits of LTCC, respectively<br>• When rare polymorphisms were included, the yield increased to 17.9%<br>• Functional expression of two *CACNA1C* mutations associated with BrS and BrS+SQTS led to loss of function in calcium channel current
**Fukuyama et al, 2013 [115]**	312 probands with LQTS, BrS, SQTS, IVF or ERS (10 with SQTS and 23 with BrS+SQTS)	Genomic DNA scanning for LTCC-related genes, *SCN5A, KCNQ1, KCNH2, KCNE1-3, KCNE5, SCN3B, KCNJ8*	Failure to identify LTCC-related variants in SQTS and BrS+SQTS cases
**Wu et al, 2015 [77]**	25 probands with SQTS and their family members (63 patients in total)	Genetic testing	• R259H-*KCNQ1* showed significantly increased current density (approximately 3-fold larger than that of wild type)<br>• R259H mutation can increase the slowly activated delayed rectifier I(Ks) in phase 3 of the cardiac action potential, which may be an infrequent cause of SQT
**Hu et al, 2017 [107]**	Probands with SQTS and family members	Clinical and genetic evaluation	• *KCNH2*-T618I was identified in 18 members of 7 unrelated families<br>• All carriers showed 100% penetrance with variable expressivity<br>• 18 members in 7 families had SCD, while 7 carriers received an ICD<br>• Quinidine was effective in prolonging QTc among 5 cases, but 3 cases still had PVCs or NSVT

			• Functional studies with *KCNE2* revealed a significant increase of I(Kr) tail-current density in homozygous and heterozygous expression
**Blancard et al, 2018 [122]**	65 patients with inherited arrhythmia syndromes (7 with SQTS)	Genetic testing for *CACNA1C* variants	No variant was detected in SQTS patients
**Christiansen et al, 2023 [121]**	34 index patients with SQTS	Genetic testing	• Potentially disease-causing variants were identified in 9 patients, mainly located in *SLC4A3*: 4 patients heterozygous for novel nonsynonymous *SLC4A3* variants-p.Arg600Cys, p.Arg621Trp, p.Glu852Asp, and p.Arg952His-and 1 patient with the known p.Arg370His variant<br>• In other SQTS genes, potentially disease-causing variants were less frequent (2× in *KCNQ1*, 1× in *KCNJ2*, and *CACNA1C* each)<br>• Dysfunction in *SLC4AE* was associated with shortened action potential of cardiomyocytes
**Harrell et al, 2015 [62]**	5 unrelated SQTS families with 3 mutations in *KCNH2* and *KCNQ1* (10 affected family members), 168 SQTS patients from previous reports	Genetic and clinical investigations	• The families are presented in the case series table (62)<br>• SQT2 exhibited a higher prevalence of bradyarrhythmia (SQT2: 6/8, 75%; non-SQT2: 5/57, 9%; p <0.001) and AF (SQT2: 5/8, 63%; non-SQT2: 12/57, 21%; p = 0.012)<br>• Of 51 mutation-positive individuals from 16 SQTS families, 9 did not manifest SQT, but exhibited other ECG abnormalities such as AF

Abbreviations: AF, atrial fibrillation; BrS, Brugada syndrome; DNA, desoxyribonucleic acid; ECG, electrocardiographic; EPS, electrophysiological study; ERS, early repolarization syndrome; ICD, implantable cardioverter defibrillator; IVF, idiopathic ventricular fibrillation; LQTS, long QT syndrome; LTCC, L-type calcium channel; NSVT, non-sustained ventricular tachycardia; PVC, premature ventricular contraction; SCD, sudden cardiac death; SQT, short QT; SQTS, short QT syndrome.

References

55. Brugada, R.; Hong, K.; Dumaine, R.; Cordeiro, J.; Gaita, F.; Borggrefe, M.; Menendez T.M.; Brugada J.; Pollevick G.D.; Wolpert C.; et al. Sudden death associated with short-QT syndrome linked to mutations in HERG. *Circulation* **2004**, *109*, 30–35.

62. Harrell, D.T.; Ashihara, T.; Ishikawa, T.; Tominaga, I.; Mazzanti, A.; Takahashi, K.; Oginosawa Y.; Abe H.; Maemura K.; Sumitomo N.; et al. Genotype-dependent differences in age of manifestation and arrhythmia complications in short QT syndrome. *Int. J. Cardiol.* **2015**, *190*, 393–402.

77. Wu, Z.J.; Huang, Y.; Fu, Y.C.; Zhao, X.J.; Zhu, C.; Zhang, Y.; Xu B.; Zhu Q.L.; Li Y. Characterization of a Chinese KCNQ1 mutation (R259H) that shortens repolarization and causes short QT syndrome 2. *J. Geriatr. Cardiol.* **2015**, *12*, 394–401.

107. Hu, D.; Li, Y.; Zhang, J.; Pfeiffer, R.; Gollob, M.H.; Healey, J.; Harrell D.T.; Makita N.; Abe H.; Sun Y.; et al. The Phenotypic Spectrum of a Mutation Hotspot Responsible for the Short QT Syndrome. *JACC Clin. Electrophysiol.* **2017**, *3*, 727–743.

115. Fukuyama, M.; Ohno, S.; Wang, Q.; Kimura, H.; Makiyama, T.; Itoh, H.; Ito M.; Horie M. L-type calcium channel mutations in Japanese patients with inherited arrhythmias. *Circ. J.* **2013**, *77*, 1799–1806.

121. Christiansen, M.K.; Kjær-Sørensen, K.; Clavsen, N.C.; Dittmann, S.; Jensen, M.F.; Guldbrandsen, H.; Pedersen L.N.; Sørensen R.H.; Lildballe D.L.; Müller K.; et al. Genetic analysis identifies the SLC4A3 anion exchanger as a major gene for short QT syndrome. *Heart Rhythm.* **2023**, *20*, 1136–1143.

122. Blancard, M.; Debbiche, A.; Kato, K.; Cardin, C.; Sabrina, G.; Gandjbakhch, E.; Probst V.; Haissaguerre M.; Extramiana F.; Hocini M.; et al. An African loss-of-function CACNA1C variant p.T1787M associated with a risk of ventricular fibrillation. *Sci. Rep.* **2018**, *8*, 14619.

136. Burashnikov, E.; Pfeiffer, R.; Barajas-Martinez, H.; Delpón, E.; Hu, D.; Desai, M.; Borggrefe M.; Hissaguerre M.; Kanter R.; Pollevick G.D.; et al. Mutations in the cardiac L-type calcium channel associated with inherited J-wave syndromes and sudden cardiac death. *Heart Rhythm.* **2010**, *7*, 1872–1882.

137. Antzelevitch, C.; Pollevick, G.D.; Cordeiro, J.M.; Casis, O.; Sanguinetti, M.C.; Aizawa, Y.; Guerchicoff A.; Pfeiffer R.; Oliva A.; Wollnik B.; et al. Loss-of-function mutations in the cardiac calcium channel underlie a new clinical entity characterized by ST-segment elevation, short QT intervals, and sudden cardiac death. *Circulation* **2007**, *115*, 442–449.
