# Supplementary material for: The Uncommon Phenomenon of Short QT Syndrome: A Scoping Review of the Literature
Source: J Pers Med. 2025 Mar 8;15(3):105. doi: 10.3390/jpm15030105 (PMC11943495; doi:10.3390/jpm15030105)
Supplement: Supplementary file 1 [file jpm-15-00105-s001.zip › Supplementary Table S6 OK.pdf]

Supplementary Table S6: Studies assessing outcomes in patients with short QT syndrome.

STUDY ID	PATIENTS	STUDY ASSESSMENTS/INTERVENTIONS	COMPARISONS	OUTCOMES
**Schimpf et al, 2003 [84]**	5 patients with SQTS	• ICD implantation due to syncope, positive family history or inducible ventricular tachyarrhythmias<br>• ICD programming	-	Inappropriate shock deliveries in 3/5 patients, 30±26 days after ICD implantation
**Giustetto et al, 2006 [111]**	29 patients with SQTS and personal and/or family history of CA, without SHD	• Clinical history<br>• Physical examination<br>• 12-lead ECG<br>• Exercise stress testing<br>• EPS<br>• Morphological evaluation<br>• Genetic analysis<br>• Median follow-up of 23 (9-49) months	-	• 18 patients (62%) were symptomatic: 10 had CA (34%) and in 8 (28%) this was the first clinical presentation<br>• CA occurred in the first months of life in 2 patients<br>• 7 patients had syncope (24%)<br>• 9 patients (31%) had palpitations with AF<br>• 14 patients received an ICD and 10 HQ prophylaxis<br>• At follow-up, 1 patient received an appropriate shock from the ICD; no patient on HQ had sudden death or syncope
**Giustetto et al, 2011 [110]**	53 patients from the European Short QT Registry	Follow-up for 64 ± 27 months	-	• Personal or family or history of CA was present in 89%<br>• SD was the clinical presentation in 32%<br>• A mutation in genes related to SQTS was found in 23% of the probands; most of them had a gain of function mutation in *HERG* (SQTS1)<br>• 24 patients received an ICD and 12 patients received long-term prophylaxis with HQ<br>• HQ was effective in preventing the induction of ventricular arrhythmias<br>• Patients with a *HERG* mutation had shorter QTc at baseline and greater QTc prolongation after treatment with HQ<br>• During follow-up: 2 symptomatic patients received appropriate ICD shocks, 1 had syncope, 3 had polymorphic NSVT; no arrhythmic events in patients receiving HQ
**Villafane et al, 2013 [80]**	25 patients with SQTS	Follow-up for 5.9 years	-	• Symptoms occurred in 14 (56%) patients (aborted SCD in 6 patients, syncope in 4 patients)<br>• Arrhythmias were common and included AF, VF, SVT and polymorphic VT<br>• 16 patients (84%) had a family or personal history of CA

				• A gene mutation associated with SQTS was identified in 5/21 probands<br>• 10 patients received medical treatment (mainly with quinidine)<br>• 11/ 25 index cases underwent ICD implantation<br>• 2 patients had appropriate ICD shocks, while inappropriate ICD shocks were observed in 64% of patients
**Mazzanti et al, 2014 [97]**	73 patients with SQTS	Follow-up for 60±14 months	-	• CA was the most frequent presenting symptom (40% of probands; range, <1 month to 41 years, male predominance); the rate of CA was 4% in the first year of life and 1.3% per year between 20 - 40 years<br>• Females had a higher risk than men<br>• The yield of genetic screening was low (14%)<br>• A history of CA was the only predictor of recurrences at follow-up<br>• 2 patterns of onset of VF were observed and were reproducible in patients with multiple occurrences of CA
**Migliore et al, 2016 [95]**	96 consecutive patients with cardiomyopathies or channelopathies (2 with SQTS) with implanted ICD	Follow-up for 72.6±53.3 months	-	• 20 patients had a total of 38 appropriate ICD interventions (4%/year)<br>• 26 patients experienced a total of 49 adverse ICD-related events (5.4%/year), namely inappropriate ICD interventions (9 patients) or device-related complications requiring surgical revision occurring (20 patients)
**Rudic et al, 2017 [86]**	62 consecutive patients with primary hereditary arrhythmia syndromes, without indication for antibradycardia therapy, with indication for ICD placement (1 patient with SQTS)	ICD implantation	-	• Subcutaneous ICD was implanted in 39 patients for secondary prevention<br>• 22 patients had a previous transvenous ICD implanted, but required revision because of infection or lead defects<br>• 20 spontaneous ventricular tachyarrhythmias requiring shock intervention occurred in 10 patients during follow-up<br>• 2 patients had inappropriate therapies due to oversensing No pocket-site infections occurred during follow-up
**El-Battrawy et al, 2019 [119]**	57 SQTS patients receiving ICD (7 families followed up in affiliated hospital and the rest from	Follow-up for a median of 67.4 months	-	• Complications during follow-up were documented in 31 (54%) patients<br>• Inappropriate shocks were seen in 33% due to T-wave oversensing (8.7%), SVT

	systematic review of the literature)			• (19%), lead failure or fracture (21%)<br>• Other complications were infection (10%), battery depletion (7%) and psychological distress (3.5%)<br>• Appropriate shocks were documented in 19%<br>• 3 patients (5%) were treated with subcutaneous ICD due to recurrent complications of transvenous ICD
**Kim et al, 2021 [102]**	34 consecutive patients with SQT from January 1999 to March 2019 from 3 university hospitals in South Korea	• Clinical and ECG features and outcomes<br>• Follow-up for 4.8 (2.0-7.8) years	Age- and sex-matched patients with normal QTc and without known cardiovascular disease	• ERP, tall T-wave, and U-wave were more frequent in patients with SQT<br>• HR was significantly slower in patients with SQT<br>• AF (11.8% vs. 2.2%, P=0.030) and VA/CA (8.7% vs. 0%, P=0.007) were significantly more frequent in patients with SQT than in those without SQT<br>• SQT was significantly associated with AF and VA/CA
**Robinson et al, 2021 [129]**	106 patients who received ICD due to CA (1 patient with SQTS)	Median follow-up of 3 years	-	• 20 patients received appropriate and 16 received inappropriate shocks during follow-up<br>• Underlying diagnosis and idiopathic status were not associated with increased incidence of appropriate shock<br>• In patients >2 years from implantation, younger age and positive exercise test were associated with appropriate shock

Abbreviations: AF, atrial fibrillation; CA, cardiac arrest; ECG, electrocardiogram; EPS, electrophysiological study; ERP, early repolarization; HQ, hydroquinidine; HR, heart rate; ICD, implantable cardioverter defibrillator; NSVT, non-sustained ventricular tachycardia; SCD, sudden cardiac death; SD, sudden death; SHD, structural heart disease; SQT, short QT; SQTS, short QT syndrome; SVT, supraventricular tachycardia; VA, ventricular arrhythmia; VF, ventricular fibrillation; VT, ventricular tachycardia.

References

80. Villafañe, J.; Atallah, J.; Gollob, M.H.; Maury, P.; Wolpert, C.; Gebauer, R.; Watanabe H.; Horie M.; Anttonen O.; Kannakeril P.; et al. Long-term follow-up of a pediatric cohort with short QT syndrome. *J. Am. Coll. Cardiol.* **2013**, *61*, 1183–1191.

85. Schimpf, R.; Antzelevitch, C.; Haghi, D.; Giustetto, C.; Pizzuti, A.; Gaita, F.; Veltmann C.; Wolpert C.; Borggrefe M. Electromechanical coupling in patients with the short QT syndrome: Further insights into the mechanoelectrical hypothesis of the U wave. *Heart Rhythm.* **2008**, *5*, 241–245.

86. Rudic, B.; Tülümen, E.; Berlin, V.; Röger, S.; Stach, K.; Liebe, V.; El-Battrawy I.; Dösch C.; Papavassiliu T.; Akin I.; et al. Low Prevalence of Inappropriate Shocks in Patients With Inherited Arrhythmia Syndromes With the Subcutaneous Implantable Defibrillator Single Center Experience and Long-Term Follow-Up. *J. Am. Heart Assoc.* **2017**, *6*, e006265.

95. Migliore, F.; Silvano, M.; Zorzi, A.; Bertaglia, E.; Siciliano, M.; Leoni, L.; De Franceschi P.; Iliceto S.; Corrado D. Implantable cardioverter defibrillator therapy in young patients with cardiomyopathies and channelopathies: A single Italian centre experience. *J. Cardiovasc. Med.* **2016**, *17*, 485–493.

97. Mazzanti, A.; Kanthan, A.; Monteforte, N.; Memmi, M.; Bloise, R.; Novelli, V.; Miceli C.; O'Rourke S.; Borio G.; Zienciuk-Krajka A.; et al. Novel insight into the natural history of short QT syndrome. *J. Am. Coll. Cardiol.* **2014**, *63*, 1300–1308.

102. Kim, D.Y.; Uhm, J.S.; Kim, M.; Kim, I.S.; Jin, M.N.; Yu, H.T.; Kim T.H.; Kim J.Y.; Joung B.; Pak H.N.; et al. Long-term prognosis of short QT interval in Korean patients: A multicenter retrospective cohort study. *BMC Cardiovasc. Disord.* **2021**, *21*, 17.

110. Giustetto, C.; Schimpf, R.; Mazzanti, A.; Scrocco, C.; Maury, P.; Anttonen, O.; Probst V.; Blanc J.J.; Sbragia P.; Dalmasso P.; et al. Long-term follow-up of patients with short QT syndrome. *J. Am. Coll. Cardiol.* **2011**, *58*, 587–595.

111. Giustetto, C.; Di Monte, F.; Wolpert, C.; Borggrefe, M.; Schimpf, R.; Sbragia, P.; Leone G.; Maury P.; Anttonen O.; Haissaguerre M.; et al. Short QT syndrome: Clinical findings and diagnostic-therapeutic implications. *Eur. Heart J.* **2006**, *27*, 2440–2447.

119. El-Battrawy, I.; Besler, J.; Ansari, U.; Liebe, V.; Schimpf, R.; Tülümen, E.; Rudic B.; Lang S.; Odening K.; Cyganek L.; et al. Long-term follow-up of implantable cardioverter-defibrillators in Short QT syndrome. *Clin. Res. Cardiol*. **2019**, *108*, 1140–1146.

129. Robinson, J.A.; Lapage, M.J.; Atallah, J.; Webster, G.; Miyake, C.Y.; Ratnasamy, C.; Ollberding N.J.; Mohan S.; Von Bergen N.H.; Johnsrude C.L.; et al. Outcomes of Pediatric Patients With Defibrillators Following Initial Presentation With Sudden Cardiac Arrest. *Circ. Arrhythmia Electrophysiol.* **2021**, *14*, E008517.
