# Supplementary material for: The Uncommon Phenomenon of Short QT Syndrome: A Scoping Review of the Literature
Source: J Pers Med. 2025 Mar 8;15(3):105. doi: 10.3390/jpm15030105 (PMC11943495; doi:10.3390/jpm15030105)
Supplement: Supplementary file 1 [file jpm-15-00105-s001.zip › Supplementary Table S7 OK.pdf]

Supplementary Table S7a. Studies assessing the effects of pharmaceutical interventions among patients with short QT syndrome.

STUDY ID	PATIENTS	STUDY ASSESSMENTS/INTERVENTIONS	COMPARISONS	OUTCOMES
**Gaita et al, 2004 [113]**	2 families with SQTS (6 patients in total)	• Drug testing (flecainide IV and per os, sotalol IV and per os, HQ per os, ibutilide IV)<br>• EPS (5/6 patients)<br>• Ventricular stimulation via the ICD while on HQ treatment<br>• 12-lead ECG	-	• Patients previously described by Gaita et al (61)<br>• Oral flecainide produced a slight QT increase, mainly due to QRS prolongation, while ibutilide (2 patients) and sotalol (3 patients) caused no changes of QT<br>• HQ was administered to 6 patients: 1 required discontinuation due to adverse effects<br>• In the remaining 5 patients, HQ caused QT prolongation, which reached the normal range<br>• Ventricular programmed stimulation was repeated via the ICD lead in 5 patients while they were on HQ treatment; in all patients, VAs were not inducible after drug administration, whereas they had been induced in 4/5 patients at basal EPS
**Wolpert et al, 2005 [78]**	1 family with SQTS (3 patients in total) with N588K mutation in *KCNH2* gene	• EPS<br>• ICD implantation<br>• Exercise testing<br>• Oral quinidine testing (2/3 patients)	10 healthy female subjects (control group)	• Family previously described by Gaita et al (61)<br>• QTpeak in V3 lead off drugs shortened less with increasing HR and the QTpeak/HR correlation was weaker than in the control group<br>• After treatment with quinidine, QTpeak decrease with increasing HR in the 2 patients treated was steeper and more strongly correlated<br>• Quinidine normalized QT at resting heart rate and rendered VF non-inducible
**Schimpf et al, 2007 [128]**	2 patients with SQTS with a N588K mutation in *HERG*	• Drug testing (disopyramide)<br>• Exercise testing<br>• Programmed ventricular stimulation via the ICD lead	-	Disopyramide increased QTc from 329 ms/QTc 315 ms, respectively, off drugs to QTc 358 ms/QTc 333 ms in both patients and restored the HR dependence of the QT
**Mazzanti et al, 2017 [96]**	17 patients with SQTS	Long-term treatment with HQ (stopped in 2 cases due to GI intolerance), for 6±1 years	-	• QTc prolongation was observed in all patients<br>• Patients on HQ experienced a reduction in both the rate of LAE from 40% to 0% and the number of LAE per patient from 0.73±0.3 to 0<br>• The annual rate of LAE in the 16 patients with a previous CA dropped from 12% before HQ to 0 on therapy
**El-Battrawy et al, 2019 [118]**	62 SQTS patients treated with antiarrhythmic drugs	Follow-up of 5.6 (1.6-7.7) years	-	• Among patients treated with HQ, QTc interval increased from 313.5±17.2 to 380.1±21.2 ms<br>• 13/41 patients suffered from at least 1 or more ventricular tachyarrhythmias before HQ initiation<br>• VAs are reduced in incidence after HQ treatment (13/41: 31% versus 3/41: 7.3%, $p < 0.001$)

Supplementary Table S7b. Studies comprising identification of short QT syndrome cases among families with history of sudden cardiac arrest or sudden cardiac death.

STUDY ID	PATIENTS	STUDY ASSESSMENTS/INTERVENTIONS	COMPARISONS	OUTCOMES
**Makarov et al, 2004 [131]**	11 children aged 3-16y from families with cases of SD at young age (<40y)	ECG analysis	-	9 of 11 children (81.1%) had SQT interval (Rautaharju formula)
**Wisten et al, 2012 [71]**	25 cases in the national Swedish study deceased from SCD during 1992–1999 with normal findings at forensic autopsy	• Blood samples obtainment from 37 first-degree relatives<br>• Mutation screening of DNA	-	• Clinical description of cases in case series table (72)<br>• 1 family with SQTS was identified
**Kumar et al, 2013 [101]**	• 109 consecutive families (411 patients) referred with 1 or more SD in the family<br>• 52 consecutive probands with UCA (and 39 relatives)	• CV assessment<br>• Genetic testing if a clinical phenotype was proven or suspected<br>• Autopsy in probands	-	• A diagnosis was made in 19 of 109 families with SADS (18%) with the majority having LQTS<br>• SQTS was diagnosed in 1 family
**Jimenez – Jalmez et al, 2015 [105]**	35 patients with UCA	• 12-lead ECG<br>• TTE<br>• Coronary angiography<br>• Pharmacologic tests (epinephrine, flecainide)<br>• Genetic testing	-	• A diagnosis was made in 18 cases (BrS: 7, CPVT: 5, LQTS: 3, ERS: 2, SQTS: 1)<br>• Pharmacologic testing was the most frequent method of diagnosis<br>• In 5 cases, the diagnosis was made based on positive genetic testing without phenotypic alterations
**Stepien – Wojno et al, 2018 [83]**	44 unrelated patients after UCA and 96 relatives	• CV assessment<br>• Next generation sequencing (31/44 patients)	-	• Diagnosis was established in 39% of probands (LQTS: 21%, SQTS: 7%, BrS: 7%, CPVT: 2%, ERS: 2%)<br>• VA was identified in the relatives of 19% of probands<br>• In 18/31 probands (54.8%), 23 rare gene variants were identified, of which only 2 were classified as pathogenic<br>• The event-free survival over a median of 4.5 years was similar in patients with or without clinical diagnosis and in carriers and noncarriers of a rare genetic variant

Supplementary Table S7c. Studies demonstrating association of short QT with other clinical conditions.

STUDY ID	PATIENTS	STUDY ASSESSMENTS/INTERVENTIONS	COMPARISONS	OUTCOMES
**Teh et al, 2007 [127]**	70 patients with epilepsy	ECG analysis	70 age-, race- and gender-matched controls	• 35 epilepsy patients and 17 matched controls had a mean QTc shorter than 0.4s<br>• Patients with cryptogenic epilepsy had a mean QTc of 0.392 ± 0.029s, significantly shorter than patients with symptomatic epilepsy
**Li et al, 2013 [100]**	150 patients with DCM	Screening for anti-*KCNQ1* autoantibodies	-	• Autoantibodies targeting *KCNQ1* were detected in 6% of study population<br>• Seropositive individuals demonstrated shorter QTc (371±39.9 ms) vs seronegative patients (408±47.9 ms)<br>• No difference in clinical severity was demonstrated between groups
**Jørgensen et al, 2015 [104]**	62 male subjects with Klinefelter syndrome	ECG analysis	62 age-matched healthy males	• Compared to controls, QTc was shorter in males with KS depending on the applied correction method<br>• QTc was shortest among testosterone-treated males with KS, while untreated had QTc interval comparable to controls<br>• No mutations in genes related to SQTS were found

Abbreviations: BrS, Brugada syndrome; CA, cardiac arrest; CPVT, catecholaminergic polymorphic ventricular tachycardia; CV, cardiovascular; DCM, dilative cardiomyopathy; DNA, desoxyribonucleic acid; ECG, electrocardiogram; EPS, electrophysiological study; ERS, early repolarization syndrome; GI, gastrointestinal; HQ, hydroquinidine; HR, heart rate; ICD, implantable cardioverter defibrillator; IV, intravenous; KS, Klinefelter syndrome; LAE, life-threatening arrhythmic events; LQTS, long QT syndrome; SADS, sudden arrhythmic death syndrome; SCD, sudden cardiac death; SD, sudden death; SQT, short QT; SQTS, short QT syndrome; SUD, sudden unexplained death; TTE, transthoracic echocardiogram; UCA, unexplained cardiac arrest; VA, ventricular arrhythmia; VF, ventricular fibrillation; VT, ventricular tachycardia.

References

71. Wisten, A.; Boström, I.M.; Mörner, S.; Stattin, E.L. Mutation analysis of cases of sudden unexplained death, 15 years after death: Prompt genetic evaluation after resuscitation can save future lives. *Resuscitation* **2012**, *83*, 1229–1234.

78. Wolpert, C.; Schimpf, R.; Giustetto, C.; Antzelevitch, C.; Cordeiro, J.; Dumaine, R.; Brugada R.; Hong K.; Bauersfeld U.; Gaita F.; et al. Further insights into the effect of quinidine in short QT syndrome caused by a mutation in HERG. *J. Cardiovasc. Electrophysiol.* **2005**, *16*, 54–58.

83. Stępień-Wojno, M.; Ponińska, J.; Rydzanicz, M.; Bilińska, M.; Truszkowska, G.; Baranowski, R.; Lutyńska A.; Biernacka E.K.; Stępińska J.; Kowalik I.; et al. Sudden cardiac arrest in patients without overt heart disease: A limited value of next generation sequencing. *Pol. Arch. Intern. Med.* **2018**, *128*, 721–730.

96. Mazzanti, A.; Maragna, R.; Vacanti, G.; Kostopoulou, A.; Marino, M.; Monteforte, N.; Bloise R.; Underwood K.; Tibollo V.; Pagan E.; et al. Hydroquinidine Prevents Life-Threatening Arrhythmic Events in Patients With Short QT Syndrome. *J. Am. Coll. Cardiol.* **2017**, *70*, 3010–3015.

100. Li, J.; Seyler, C.; Wiedmann, F.; Schmidt, C.; Schweizer, P.A.; Becker, R.; Katus H.A.; Thomas D. Anti-KCNQ1 K^+ channel autoantibodies increase IKs current and are associated with QT interval shortening in dilated cardiomyopathy. *Cardiovasc. Res.* **2013**, *98*, 496–503.

101. Kumar, S.; Peters, S.; Thompson, T.; Morgan, N.; Maccicoca, I.; Trainer, A.; Zentner D.; Kalman J.M.; Winship I.; Vohra J.K. Familial cardiological and targeted genetic evaluation: Low yield in sudden unexplained death and high yield in unexplained cardiac arrest syndromes. *Heart Rhythm.* **2013**, *10*, 1653–1660.

104. Jørgensen, I.N.; Skakkebaek, A.; Andersen, N.H.; Pedersen, L.N.; Hougaard, D.M.; Bojesen, A.; Trolle C.; Gravholt C.H. Short QTc interval in males with klinefelter syndrome-influence of CAG repeat length, body composition, and testosterone replacement therapy. *Pacing Clin. Electrophysiol.* **2015**, *38*, 472–482.

105. Jiménez-Jáimez, J.; Peinado, R.; Grima, E.Z.; Segura, F.; Moriña, P.; Sánchez Muñoz, J.J.; Mazuelos F.; Cózar R.; Gimeno J.R.; Heras R.P.; et al. Diagnostic Approach to Unexplained Cardiac Arrest (from the FIVI-Gen Study). *Am. J. Cardiol.* **2015**, *116*, 894–899.
113. Gaita, F.; Giustetto, C.; Bianchi, F.; Schimpf, R.; Haissaguerre, M.; Calò, L.; Brugada R.; Antzelevitch C.; Borggrefe M.; Wolpert C. Short QT syndrome: Pharmacological treatment. *J. Am. Coll. Cardiol.* **2004**, *43*, 1494–1499.
118. El-Battrawy, I.; Besler, J.; Li, X.; Lan, H.; Zhao, Z.; Liebe, V.; Schimpf R.; Lang S.; Wolpert C.; Zhou X.; et al. Impact of Antiarrhythmic Drugs on the Outcome of Short QT Syndrome. *Front. Pharmacol*. **2019**, *10*, 771.
127. Teh, H.S.; Tan, H.J.; Loo, C.Y.; Raymond, A.A. Short QTc in epilepsy patients without cardiac symptoms. *Med. J. Malays.* **2007**, *62*, 104–108.
128. Schimpf, R.; Veltmann, C.; Giustetto, C.; Gaita, F.; Borggrefe, M.; Wolpert, C. In vivo effects of mutant HERG K+ channel inhibition by disopyramide in patients with a short QT-1 Syndrome: A pilot study. *J. Cardiovasc. Electrophysiol.* **2007**, *18*, 1157–1160.
131. Makarov, L.M.; Chuprova, S.N.; Kiseleva, I.I. QT interval shortening in families with history of sudden death at young age. *Kardiologiia* **2004**, *44*, 51–56.
