# Supplementary material for: The Uncommon Phenomenon of Short QT Syndrome: A Scoping Review of the Literature
Source: J Pers Med. 2025 Mar 8;15(3):105. doi: 10.3390/jpm15030105 (PMC11943495; doi:10.3390/jpm15030105)
Supplement: Supplementary file 1 [file jpm-15-00105-s001.zip › Supplementary Table S8 OK.pdf]

Supplementary Table S8. Studies involving implementation of various diagnostic modalities among patients with short QT syndrome.

STUDY ID	PATIENTS	STUDY ASSESSMENTS/INTERVENTIONS	COMPARISONS	OUTCOMES
**Anttonen et al, 2008 [52]**	1 family with SQTS (3 patients in total)	• 24h ECG Holter recordings<br>• TTE	9 healthy controls	• Patients are described in the case series table (51)<br>• SQTS patients have increased and autonomically uncontrolled ECG transmural dispersion of repolarization<br>• According to experimental models, the results may in part explain increased vulnerability of SQTS patients to VAs
**Schimpf et al, 2008 [85]**	5 SQTS patients from 2 unrelated families with a history of SCD	• TTE<br>• 12-lead ECG	5 age-matched and gender-matched controls	• Patients previously described by Gaita et al (60)<br>• QT intervals were 268+/-18 ms (QTc 285+/-28 ms) in SQTS vs 386+/-20 ms (QTc 420+/-22 ms) in controls (P<0.005)<br>• The interval from AV closure to the beginning of the U-wave was 8 +/- 4 ms in patients and 15 +/- 11 ms in controls (P=0.25)<br>• The inscription of the U-wave in SQTS patients coincided with AV closure and isovolumic relaxation, supporting the hypothesis that the U-wave is related to mechanical stretch
**Anttonen et al, 2009 [124]**	3 families (10 patients in total) with SQT	T-wave morphology assessed using the principal component analysis	149 healthy controls	• QTc was significantly shorter and T-wave amplitude in lead V2 higher in the SQT subjects compared to healthy controls (P<0.001 for both)<br>• The TCRT was markedly abnormal among the symptomatic patients with SQTS<br>• None of the 3 asymptomatic patients with SQT but without a history of arrhythmic events had an abnormally low TCRT
**Anttonen et al, 2009 [138]**	• 10 patients with SQTS with documented VT or aborted SCD<br>• 12 patients with SQTS with no documented VT, SCD or other arrhythmias	ECG analysis	Age- and gender-matched control group with normal QT (20 patients)	• All patients with symptomatic SQTS had shorter Jpoint-Tpeak interval than event-free subjects with a SQT on ECG or healthy subjects with a normal QT<br>• The rate-corrected Tpeak-Tend c/QTc ratio was significantly higher among SQTS patients compared to the other 2 groups

**Watanabe et al, 2010 [79]**	• SQTS cohort (N = 37)<br>• SQTS cohort with arrhythmic events - SQT control cohort (N = 44)	ECG analysis	Control cohort with normal QT interval (N=185)	• HR, PR and QRS duration were similar among the 3 cohorts<br>• ERP was more common in the SQTS cohort (65%) than in the SQT control cohort (30%) and the normal QT control cohort (10%)<br>• Duration from T-wave peak to T-wave end was longer in SQTS cohort than in the SQT control cohort, although QT and QTc intervals were similar<br>• In multivariate models, ERP was associated with arrhythmic events in the SQTS cohort<br>• ECG parameters including QT and QTc were not associated with arrhythmic events in the SQTS cohort
**Maury et al, 2012 [98]**	13 patients with SQTS	Microwolt TWA assessment using spectral analysis	-	• TWA was negative in all but one patient<br>• Patients with previous SD displayed shorter QT and higher resting HR compared to the remaining cases
**Tülümen et al, 2014 [81]**	64 patients with SQTS	Digitalized 12-lead ECGs analyzed for PQD in all leads and for QT in leads II and V5	-	• PQD was seen in 265 (35%) leads from 52 (81%) patients and was more frequent in leads II, V3, aVF, V4, and I<br>• 9/64 (14%) patients presented with atrial tachyarrhythmias, and all of them had PQD
**Frea et al, 2015 [116]**	15 patients with SQTS<br>• 7 with *HERG* mutation<br>• 3 with *KCNQ1* mutation	• 12-lead ECG<br>• TTE	Age and sex-matched controls	• SQTS patients showed reduced LV contraction (GLS: -16%±3.4% vs -22.6%±1.7%, P<0.00; MPI 0.59±0.17 vs 0.34±0.08, P<0.001) and a higher incidence of EF <55% compared to controls<br>• Mechanical dispersion assessed by TDI (P<0.01) and STE (P<0.001) was higher in the SQTS group than in controls<br>• Each parameter showed a significant inverse correlation with QT interval but not with QT dispersion
**Giustetto et al, 2015 [109]**	21 patients with SQTS	Exercise test	20 matched controls	• Rest and peak exercise HR were not different in the 2 groups<br>• SQTS patients showed shorter QT compared with controls both at rest (276±27 ms vs. 364±25 ms, P<0.0001) and at peak exercise (228±27 ms vs. 245±26 ms, P=0.05)<br>• Regression analysis of QT/HR relationship revealed a less steep slope

				for SQTS patients compared with the control group
**Suzuki et al, 2021 [82]**	34 young patients with SQTS	ECG analysis	61 apparently healthy subjects with QTc<360 ms who were selected from 13,314 participants in a school-based screening program	• QT, QTcB, QTcF, J-Tpeak, J-Tpeak cB, and J-Tpeak cF were significantly shorter in the SQTS group than in the control group<br>• On ROC curve, the AUC was largest for QTcB (0,888) among QT, QTcB, and QTcF, with a cut-off value of 316 ms (sensitivity: 79.4% and specificity: 96.7%)<br>• The AUC was largest for J-Tpeak cB (0.848) among J-Tpeak, J-Tpeak cB, and J-Tpeak cF, with a cut-off value of 181 ms (sensitivity: 80.8% and specificity: 91.8%)<br>• ERP was found more frequently in the SQTS group than in the control group (67% vs. 23%, P=0.001)
**Extramiana et al, 2008 [117]**	27 subjects from a single family with SQTS<br>• Based on QTc (Bazett's formula), 4 men were considered to have SQTS (QTc <or=340 ms), and 16 were classified to have "suspected" SQTS (QT <or=320 ms and/or QTc <or=380 ms)	• 12 lead ECG<br>• Laboratory testing<br>• TTE<br>• 24h ECG Holter monitoring	62 healthy controls	• Patients with SQTS and controls had lower coefficients of the QT/RR relation when compared with Bazett and Fridericia formulas<br>• The QT rate-dependence was lower in patients with SQTS (a 0.146+/-0.07) when compared with controls (a 0.203+/-0.039, p<0.05)<br>• The universal correction-formulas provided higher values of corrected QT duration at the mean Holter HR than a subject-specific correction formula (p <0.05), which induced false negative diagnoses

Abbreviations: AUC, area under the curve; AV, aortic valve; CI, confidence interval; ECG, electrocardiogram; EF, ejection fraction; ERP, early repolarization; GLS, global longitudinal strain; HR, heart rate; ICD, implantable cardioverter defibrillator; LV, left ventricular; MPI, myocardial performance index; OR, odds ratio; PQD, PQ depression; QTcB, QT correction using Bazett's formula; QTcF, QT correction using Fridericia's formula; ROC, receiver operating characteristic; SCD, sudden cardiac death; SD, sudden death; SQT, short QT; SQTS, short QT syndrome; STE, speckle tracking echocardiography; TCRT, total cosine of the angle between the main vectors of the QRS and T-wave loops; TDI, tissue Doppler imaging; TTE, transthoracic echocardiogram; TWA, T-wave alternans; VA, ventricular arrhythmia; VF, ventricular fibrillation; VT, ventricular tachycardia.

References

52. Anttonen, O.; Väänänen, H.; Junttila, J.; Huikuri, H.V.; Viitasalo, M. Electrocardiographic transmural dispersion of repolarization in patients with inherited short QT syndrome. *Ann. Noninvasive. Electrocardiol*. **2008**, *13*, 295–300.

79. Watanabe, H.; Makiyama, T.; Koyama, T.; Kannankeril, P.J.; Seto, S.; Okamura, K.; Oda H.; Itoh H.; Okada M.; Tanabe N.; et al. High prevalence of early repolarization in short QT syndrome. *Heart Rhythm.* **2010**, *7*, 647–652.

81. Tülümen, E.; Giustetto, C.; Wolpert, C.; Maury, P.; Anttonen, O.; Probst, V.; Blanc J.J.; Sbragia P.; Scrocco C.; Rudic B.; et al. PQ segment depression in patients with short QT syndrome: A novel marker for diagnosing short QT syndrome? *Heart Rhythm.* **2014**, *11*, 1024–1030.

82. Suzuki, H.; Horie, M.; Ozawa, J.; Sumitomo, N.; Ohno, S.; Hoshino, K.; Ehara E.; Takahashi K.; Maeda Y.; Yoshinaga M.; et al. Novel electrocardiographic criteria for short QT syndrome in children and adolescents. *Europace* **2021**, *23*, 2029–2038.

85. Schimpf, R.; Antzelevitch, C.; Haghi, D.; Giustetto, C.; Pizzuti, A.; Gaita, F.; Veltmann C.; Wolpert C.; Borggrefe M. Electromechanical coupling in patients with the short QT syndrome: Further insights into the mechanoelectrical hypothesis of the U wave. *Heart Rhythm.* **2008**, *5*, 241–245.

98. Maury, P.; Extramiana, F.; Giustetto, C.; Cardin, C.; Rollin, A.; Duparc, A.; Mondoly P.; Denjoy I.; Delay M.; Messali A.; et al. Microvolt T-wave alternans in short QT syndrome. *Pacing Clin. Electrophysiol.* **2012**, *35*, 1413–1419.

109. Giustetto, C.; Scrocco, C.; Schimpf, R.; Maury, P.; Mazzanti, A.; Levetto, M.; Anttonen O.; Dalmasso P.; Cerrato N.; Gribaudo E.; et al. Usefulness of exercise test in the diagnosis of short QT syndrome. *Europace* **2015**, *17*, 628–634.

116. Frea, S.; Giustetto, C.; Capriolo, M.; Scrocco, C.; Fornengo, C.; Benedetto, S.; Bianchi F.; Pidello S.; Morello M.; Gaita F. New echocardiographic insights in short QT syndrome: More than a channelopathy? *Heart Rhythm.* **2015**, *12*, 2096–2105.

117. Extramiana, F.; Maury, P.; Maison-Blanche, P.; Duparc, A.; Delay, M.; Leenhardt, A. Electrocardiographic biomarkers of ventricular repolarisation in a single family of short QT syndrome and the role of the Bazett correction formula. *Am. J. Cardiol.* **2008**, *101*, 855–860.

124. Anttonen, O.; Junttila, J.; Giustetto, C.; Gaita, F.; Linna, E.; Karsikas, M.; Seppänen T.; Perkiömäki J.S.; Mäkikallio T.H.; Brugada R.; et al. T-Wave morphology in short QT syndrome. *Ann. Noninvasive. Electrocardiol.* **2009**, *14*, 262–267.

138. Anttonen, O.; Junttila, M.J.; Maury, P.; Schimpf, R.; Wolpert, C.; Borggrefe, M.; Giustetto C.; Gaita F.; Sacher F.; Haissaguerre M.; et al. Differences in twelve-lead electrocardiogram between symptomatic and asymptomatic subjects with short QT interval. *Heart Rhythm.* **2009**, *6*, 267–271.
