# Supplementary material for: The Uncommon Phenomenon of Short QT Syndrome: A Scoping Review of the Literature
Source: J Pers Med. 2025 Mar 8;15(3):105. doi: 10.3390/jpm15030105 (PMC11943495; doi:10.3390/jpm15030105)
Supplement: Supplementary file 1 [file jpm-15-00105-s001.zip › Supplementary Table S9 OK.pdf]

Supplementary Table S9. Studies involving other advances in research regarding short QT syndrome.

STUDY ID	PATIENTS	STUDY ASSESSMENTS/INTERVENTIONS	COMPARISONS	OUTCOMES
**Viskin et al, 2004 [126]**	28 patients with idiopathic VF	ECG analysis	270 age- and gender-matched controls	• QTc of males with idiopathic VF was shorter than QTc of healthy males (371±22 ms vs 385±19 ms, P=0.034)<br>• SQT was found frequently among males with idiopathic VF<br>• No such differences were apparent among women
**Rollin et al, 2017 [87]**	16 patients with SQTS	Standard EPS	15 controls with similar clinical characteristics	• Atrial RP were significantly shorter in SQTS compared with controls at 600- and 500-ms basic cycle lengths<br>• Baseline ventricular RP were significantly shorter in SQTS patients than in controls, both at the apex and RVOT and for any cycle length<br>• A cut-off value of baseline RP<200 ms at the RVOT either at 600- or 500-ms cycle length had a sensitivity of 86% and a specificity of 100% for the diagnosis of SQTS
**Gavey et al, 2018 [112]**	8 patients having at least 2 episodes of resuscitated VA and having undergone CSD (1 patient with SQTS)	Follow-up	-	• Affected persons previously described by Gaita et al (60)<br>• All patients underwent left CSD, 2 subsequently required right CSD<br>• 4/6 ICD patients improved dramatically<br>• 2 patients non-compliant with medical therapy had no significant improvement
**Pasero et al, 2023 [91]**	104 patients with SQTS<br>• 37 with a documented major arrhythmic event at presentation and/or during follow-up	ECG analysis with the aid of AI algorithms<br>• 13 ECG features were measured independently by 3 expert cardiologists; then, the dataset was randomly divided into 3 subsets (training, validation, and testing)<br>• 5 shallow neural networks were trained, validated, and tested to predict subject-specific class (non-event/event) using different subsets of ECG features<br>• Deep learning and machine learning algorithms and logistic regression were trained, validated, and tested directly on the scanned ECG images<br>• A shallow neural network, a 1-D transformer classifier, and a 1-D CNN were trained, validated, and tested on ECG signals extracted from the scanned images	-	• AI can help clinicians in better stratifying risk of arrhythmia in patients with SQTS<br>• Shallow neural networks' processing features showed the best performance in identifying patients that will not suffer from a potentially lethal event

Abbreviations: AI, artificial intelligence; CNN, convolutional neural network; CSD, cardiac sympathetic denervation; ECG, electrocardiogram; EPS, electrophysiological study; ICD, implantable cardioverter defibrillator; RP, refractory period; RVOT, right ventricular outflow tract; SQT, short QT; SQTS, short QT syndrome; VA, ventricular arrhythmia; VF, ventricular fibrillation.

References

87. Rollin, A.; Gandjbakhch, E.; Giustetto, C.; Scrocco, C.; Fourcade, C.; Monteil, B.; Mondoly P.; Cardin C.; Maupain C.; Gaita F.; et al. Shortening of the Short Refractory Periods in Short QT Syndrome. *J. Am. Heart Assoc.* **2017**, *6*, e005684.
91. Pasero, E.; Gaita, F.; Randazzo, V.; Meynet, P.; Cannata, S.; Maury, P.; Giustetto C. Artificial Intelligence ECG Analysis in Patients with Short QT Syndrome to Predict Life-Threatening Arrhythmic Events. *Sensors* **2023**, *23*, 8900.
112. Garvey, E.M.; Papez, A.L.; Notrica, D.M.; Egan, J.C.; Molitor, M.; Cohen, M.I.; van Leeuwen K. Thoracoscopic Cardiac Sympathetic Denervation: Adjunct Therapy for Secondary Prevention of Life-Threatening Ventricular Arrhythmias in Children. *J. Laparoendosc. Adv. Surg. Tech. A* **2018**, *28*, 1387–1392.
126. Viskin, S.; Zeltser, D.; Ish-Shalom, M.; Katz, A.; Glikson, M.; Justo, D.; Tekes-Manova D.; Belhassen B. Is idiopathic ventricular fibrillation a short QT syndrome? Comparison of QT intervals of patients with idiopathic ventricular fibrillation and healthy controls. *Heart Rhythm.* **2004**, *1*, 587–591.
